# RNA-Seq Analysis Reveals Genes Related to Photoreception, Nutrient Uptake, and Toxicity in a Noxious Red-Tide Raphidophyte *Chattonella antiqua*

**DOI:** 10.3389/fmicb.2019.01764

**Published:** 2019-07-31

**Authors:** Tomoyuki Shikata, Fumio Takahashi, Hiroyo Nishide, Shuji Shigenobu, Yasuhiro Kamei, Setsuko Sakamoto, Kouki Yuasa, Yoshitaka Nishiyama, Yasuhiro Yamasaki, Ikuo Uchiyama

**Affiliations:** ^1^National Research Institute of Fisheries and Environment of Inland Sea, Fisheries Research and Education Agency, Hatsukaiti, Japan; ^2^Department of Biotechnology, College of Life Sciences, Ritsumeikan University, Kusatsu, Japan; ^3^Japan Science and Technology Agency, Precursory Research for Embryonic Science and Technology, Kawaguchi, Japan; ^4^Laboratory of Genome Informatics, National Institute for Basic Biology, National Institutes of Natural Sciences, Okazaki, Japan; ^5^Core Research Facilities, National Institute for Basic Biology, Okazaki, Japan; ^6^Department of Biochemistry and Molecular Biology, Graduate School of Science and Engineering, Saitama University, Sakura-ku, Japan; ^7^Laboratory of Environmental Biology, Department of Applied Aquabiology, National Fisheries University, Fisheries Research and Education Agency, Yamaguchi, Japan

**Keywords:** aureochrome, cryptochrome, harmful algae, NADPH-oxidase, phytochrome, pigment biosynthesis, transcriptome, transporter

## Abstract

Aquaculture industries are under threat from noxious red tides, but harm can be mitigated by precautions such as early harvesting and restricting fish feeding to just before the outbreak of a red tide. Therefore, accurate techniques for forecasting red-tide outbreaks are strongly needed. Omics analyses have the potential to expand our understanding of the eco-physiology of these organisms at the molecular level, and to facilitate identification of molecular markers for forecasting their population dynamics and occurrence of damages to fisheries. Red tides of marine raphidophytes, especially *Chattonella* species, often extensively harm aquaculture industries in regions with a temperate climate around the world. A red tide of *Chattonella* tends to develop just after an input of nutrients along the coast. *Chattonella* displays diurnal vertical migration regulated by a weak blue light, so it photosynthesizes in the surface layer during the daytime and takes up nutrients in the bottom layer during the nighttime. Superoxide produced by *Chattonella* cells is a strong candidate for the cause of its toxicity to bacteria and fishes. Here we conducted mRNA-seq of *Chattonella antiqua* to identify genes with functions closely related to the dynamics of the noxious red tide, such as photosynthesis, photoreception, nutrient uptake, and superoxide production. The genes related to photosynthetic pigment biosynthesis and nutrient uptake had high similarity with those of model organisms of plants and algae and other red-tide microalgae. We identified orthologous genes of photoreceptors such as aureochrome (newly five genes), the cryptochrome/photolyase (CRY/PHR) family (6-4PHR, plant CRY or cyclobutane pyrimidine dimer [CPD] Class III, CPD Class II, and CRY-DASH), and phytochrome (four genes), which regulate various physiological processes such as flagellar motion and cell cycle in model organisms. Six orthologous genes of NADPH oxidase, which produces superoxide on the cell membrane, were found and divided into two types: one with 5–6 transmembrane domains and another with 11 transmembrane domains. The present study should open the way for analyzing the eco-physiological features of marine raphidophytes at the molecular level.

## Introduction

Red tides sometimes impact fish and shellfish aquaculture industries and tourism in coastal waters around the world. Long-term studies have provided information on taxonomy, life cycle, and eco-physiology of organisms that cause harmful red tides, but no practical method to control the causative microorganisms has been established (Anderson, 1997). On the other hand, precautionary field interventions such as early harvesting, restricting fish feeding, and transport of fish cages into zones free of red tides have shown efficacy in mitigating the damage (Kim, 2006). These interventions require monitoring of the distribution of noxious flagellates and forecasting the occurrence of a red tide; however, development of forecasting techniques based on environmental factors by using numerical and statistical models has been difficult because the mechanism underlying the development of red tides remains unknown (Glibert et al., 2010).

Understanding the molecular mechanism of red-tide formation should lead to development of molecular markers that forecast the impact and its toxicity. However, molecular analyses require genetic information. Recently, whole transcriptome analysis by total mRNA-seq has been used to rapidly accumulate genetic information and data on physiological molecular processes in noxious red-tide organisms (Ryan et al., 2014; Kimura et al., 2015; Guo et al., 2016). For non-model organisms with large genome sizes, the large cost and effort required to sequence the whole genome may make total mRNA-seq analysis a better alternative for obtaining genetic information.

Marine raphidophytes are stramenopiles, and most of them cause noxious red tides in temperate coastal waters around the world. *Heterosigma akashiwo* sometimes forms dense red tides that cause harm to fish aquaculture industries around the world (Smayda, 1998). In Japan, the genus *Chattonella*, including *C.antiqua*, *C. marina*, and *C. ovata*, has caused the most harm to the aquaculture industry (Imai and Yamaguchi, 2012). *Chattonella subsalsa* forms red tides in the United States and Europe (Zhang et al., 2006; Satta et al., 2017) but not in Japan.

Red tides of *H. akashiwo* and *Chattonella* spp. tend to occur frequently in eutrophic areas (Imai et al., 2006) or just after an influx of nutrients (Nakamura and Watanabe, 1983; Nakamura et al., 1989; Shikata et al., 2008), although the nutrient requirement of *C. antiqua* is not higher than that of other red-tide flagellates (Nakamura, 1985). Information on pathways and enzymes related to uptake and metabolisms of nutrients may resolve this apparent discrepancy. *Heterosigma akashiwo* and *C. antiqua* undergo diurnal vertical migrations that enable them to photosynthesize in the surface layers during the daytime and take up nutrients in bottom layers during the nighttime (Watanabe et al., 1991). The rhythm of diurnal vertical migration is reset by light within a specific range of wavelengths (Shikata et al., 2013). Moreover, negative phototaxis and positive geotaxis are also induced by light with different wavelengths (Shikata et al., 2016). Therefore, the motility can be regulated by photoreceptors that have unique spectra for absorbance and action. The gene encoding a blue light receptor aureochrome has been isolated in *H. akashiwo* and *C. antiqua* (Ishikawa et al., 2009; Ji et al., 2017), but there is little information on the genes encoding other photoreceptors such as a UV/blue light receptor, cryptochrome (CRY) and photolyase (PHR), and a red/far-red light receptor, phytochrome, which function in physiological control in various organisms (Ahmad and Cashmore, 1993; Furuya, 1993; Smith, 2000; Lin and Todo, 2005).

Ichthyotoxicity of marine raphidophytes, especially *Chattonella*, has also been energetically studied. The main hypothesis is that the fish die from lack of oxygen due to gill lesions and induction of excessive mucus production from gill cells by *Chattonella* cells (Ishimatsu et al., 1996). Because *Chattonella* cells produce large quantities of superoxide known as one of reactive oxygen species (ROS) compared to other phytoplankters (Oda et al., 1997), superoxide is suspected as a factor contributing to gill damage. Moreover, *C. marina* strongly inhibits marine bacteria proliferation, but the toxic effect is completely suppressed by the addition of catalase and superoxide dismutase (Oda et al., 1992). Therefore, the ability to produce superoxide may enable *Chattonella* to outcompete other microorganisms. However, there is little information on molecules and enzymes related to superoxide generation in *Chattonella*, although it is well known that NADPH oxidase (NOX) produces superoxide in membranes of cells and phagosome in plants and animals (Canton and Grinstein, 2014).

Here, we sequenced mRNA of *C. antiqua*, which frequently blooms and causes tremendous damage to the aquaculture industry in Japan, to seek genes closely related to development and toxicity of a red tide: e.g., those involved in photosynthesis, photoreception, nutrient uptake, and superoxide production. We then compared the data with sequences of model organisms, such as *Arabidopsis* and *Chlamydomonas*, noxious red-tide dinoflagellates in Japan (*Karenia mikimotoi*, *Heterocapsa circularisquama*) and other raphidophyte species (*H. akashiwo*, *C. subsalsa*).

## Materials and Methods

### Culture Conditions

We used a clonal axenic strain of *C. antiqua* (3KGY, Shikata et al., 2015, 2016) isolated from the Yatsushiro Sea, Japan. Cultures were maintained in 50-mL Erlenmeyer flasks containing 25 mL of modified SWM-3 medium (Chen et al., 1969; Shikata et al., 2011) with salinity of 30 at 25°C under 200 μmol photons m^–2^ s^–1^ of white fluorescent light illumination (FL20SW, Toshiba Lighting and Technology Corporation, Kanagawa, Japan) on a 12 h:12 h light:dark cycle (light period: local time [LT] 0600–1800). The photon flux density was measured with a Quantum Scalar Laboratory Irradiance Sensor (QSL-2101, Biospherical Instruments Inc., San Diego, CA, United States). During exponential growth (10,000 cells mL^–1^), *C. antiqua* cells were harvested during the daytime (LT 1100–1200, *n* = 3 flasks) or nighttime (LT 1900–2000, *n* = 3 flasks).

We also used a clonal axenic strain of *H. circularisquama* (N-30) isolated from Asoura, Japan; *H. circularisquama* is a dinoflagellate that forms a red tide that kills shellfish, specifically. The cultures were maintained under the same conditions as *C. antiqua*, and cell harvesting was conducted during the daytime (LT 1100–1200, *n* = 1 flask).

### RNA Extraction and cDNA Library

First, 25 mL of *C. antiqua* culture or 50 mL of *H. circularisquama* culture were concentrated to 5 mL by using a Nuclepore filter (pore diameter, 3 μm; Whatman, Kent, United Kingdom). Shortly thereafter, 5 mL of RNase inhibitor (47.5 mL of ethanol plus 2.68 g phenol) were added to the cell suspension and vigorously mixed. Cell pellets were obtained by centrifugation at 2,500 *g* for 5 min at room temperature and stored at −80°C until RNA extraction. Total RNA was isolated from the cell pellets and purified using RNeasy Plant Mini kits (QIAGEN, Valencia, CA, United States). The quantity and quality of total RNA was determined using an automated electrophoresis system, ExperionTM (Bio-Rad Laboratories, Hercules, CA, United States). cDNA libraries for *C. antiqua* and *H. circularisquama* were constructed by using an Illumina Gene Expression Sample Prep Kit (Illumina Inc., San Diego, CA, United States). Sequencing was conducted using an Illumina HiSeq 2000 system (Illumina Inc., San Diego, CA, United States).

### Assembly and Expression Analysis

Adapters and low-quality bases were removed from the raw data by using Cutadapt (Martin, 2011). The combined reads of all six samples of *C. antiqua* as well as the reads of *H. circularisquama* were assembled using Trinity software (Grabherr et al., 2011). The original reads of *C. antiqua* samples were mapped onto the contigs by using Bowtie2 (Langmead and Salzberg, 2012), and then the abundance of each contig was estimated using RSEM (Li and Dewey, 2011). The degree of differential gene expression between day and night samples was evaluated using EdgeR (Robinson et al., 2010). The raw read transcriptome sequences were submitted to the DDBJ Sequence Read Archive database under BioProject IDs PRJDB7469 and PRJDB7513. The assembled and annotated sequence data can be obtained from the web site http://hab.nibb.ac.jp.

### Gene Annotation

For each contig of *C. antiqua*, coding sequences (CDS) were extracted using TransDecoder^1^. Similarity searches were conducted using BLASTX (criterion, *E* -value < 0.001) against the NCBI non-redundant protein (NR) database to extract top hits. Motifs and domains were searched using InterProScan (Jones et al., 2014). Gene ontologies (GOs) were assigned to each contig with the default parameter setting (*E* -value < 10^–6^), and the Fisher’s exact test implemented in Blast2go (Conesa et al., 2005) was used to identify significantly enriched GO terms in each differentially expressed gene set.

### Comparative Genome/Transcriptome Analysis

The translated gene sets of *C. antiqua* and *H. circularisquama* were compared with those from three existing RNA-seq datasets of harmful algae and 10 existing genome sequences of various algae and a plant model organism, *Arabidopsis thaliana* (Supplementary Table S1). RNA sequence data of *C. subsalsa* and *H. akashiwo* were obtained from the NCBI Sequence Read Archive database (accession numbers, SRR1300240 and SRR1296916, respectively) and assembled using Trinity software. Contig sequences constructed from the RNA sequence of *K. mikimotoi* were obtained from the authors (Kimura et al., 2015). Coding sequences were extracted from the above *K. mikimotoi* contig data, and the contig data for *C. antiqua* and *H. circularisquama* (obtained in this study) by using TransDecoder software. For comparative analysis, draft genome sequences of the following microorganisms and algae—*Emiliania huxleyi*, *Cyanidioschyzon merolae*, *Phaeodactylum tricornutum*, *Thalassiosira pseudonana*, *Nannochloropsis gaditana*, *Phytophthora sojae*, *Saprolegnia diclina*, and *Ectocarpus siliculosus*—and complete genome sequences of *A. thaliana* and *Chlamydomonas reinhardtii*, were obtained from the NCBI. The DomClust program (Uchiyama, 2006) implemented in the RECOG system (Uchiyama, 2017)^2^ was used to identify orthologous relationships among the proteins identified in the above genomes and transcriptomes. RECOG was also used to manage and visualize the data obtained from comparative analysis among *C. antiqua* and the other organisms in the search for orthologous genes related to photoreception, nutrient uptake, and superoxide production.

### Phylogenetic Analysis

We inferred species phylogeny among the above organisms based on the concatenated protein sequence of the conserved and nearly one-to-one orthologs. For this purpose, we extracted orthologous groups (OGs) containing at least one or two genes for each genome and at least one gene for each transcriptome (we considered no upper limit for transcriptome data because they generally contain multiple isoforms from a single gene, which can generate more apparent “paralogs” than reality). We selected the longest sequence if multiple genes existed in the organism. Multiple sequence alignments were then constructed using MAFFT (Katoh and Standley, 2013), conserved blocks were extracted using Gblocks (Castresana, 2000), and a phylogenetic tree was drawn based on the concatenated conserved block by using FastTree (Price et al., 2010).

We also conducted phylogenetic analyses on some orthologous genes related to photoreception, nutrient uptake, and superoxide production. The amino acid sequences of typical domains and motifs were aligned using ClustalX 2.1 with the default options (Thompson et al., 1997). Neighbor-Joining (NJ) trees were constructed with ClustalX 2.1 and MEGA software (Kumar et al., 2016). Maximum-likelihood (ML, Guindon and Gascuel, 2003) and most parsimony (MP) trees were determined using MEGA, based on various models; bootstrap analysis for NJ trees (1,000 replications), ML and NP trees (100 replications) was performed.

## Results

### Sequencing, *de novo* Assembly, and Annotation

RNA sequencing generated 186 million paired reads (2 × 101 bp) from daytime and nighttime *C. antiqua* cDNA libraries, yielding assembled sequences consisting of 67,823 contigs with a mean length of 1,091 bp and N50 of 1,838 bp. A total of 45.6 million paired reads (2 × 101 bp) were also generated from the *H. circularisquama* library, yielding assembled sequences consisting of 195,802 contigs with a mean length of 698 bp and N50 of 997 bp. The average GC contents of *C. antiqua* and *H. circularisquama* samples were 41.9 and 63.1%, respectively (Table 1).

**TABLE 1:** Comparison of parameters in mRNA-seq among *Chattonella antiqua* and the other harmful flagellates.

**Species name**	Read count (million reads)	Contig or gene number	**N-50**	Mean length (bp)	GC content (%)	Annotation to NR (%)	**References**
*Chattonella antiqua*	371	67,823	1,838	1,091	42	38	This study
*Chattonella subsalsa*	33	41,237	1,274	799	42	–	This study
*Heterosigma akashiwo*	26	45,293	755	579	52	–	This study
*Heterocapsa circularisquama*	44	113,481	997	674	63	–	This study
*Alexandrium catenella*	242	155,353	1,549	1,003	61	30	Zhang et al., 2014
*Cochlodinium polykrikoides*	173	191,212	1,550	922	–	16–63	Guo et al., 2016
*Karenia mikimotoi*	10	153,406	900	687	52	–	Kimura et al., 2015
*Karenia brevis*	51–78	84,309–93,668	2,529	1,552	–	43–45	Ryan et al., 2014

TransDecoder identified 39,031 CDSs among the contigs of *C. antiqua* samples. Each CDS was then searched against the NCBI NR protein sequence database by using BLASTP with an *e* -value cutoff of 10^–3^, resulting in annotation of 25,808 CDSs (top-hit entries), of which 55% belonged to stramenopiles (Supplementary Figure S1).

GO analysis using GO slim (Gene Ontology Consortium, 2004) was performed to classify the predicted functions of *C. antiqua* transcripts. Of the 39,031 CDSs identified, 4,966 (13%) were assigned with one or more GO terms in the biological process category, 3,854 (10%) in the cellular component category, and 6,006 (15%) in the molecular function category.

To evaluate the completeness of the gene set obtained from *C. antiqua* transcriptome analysis, we conducted Benchmarking Universal Single-Copy Orthologs (BUSCO) analysis (Simao et al., 2015). Of the 303 BUSCOs in the “eukaryota_odb9” dataset, 93.4% were complete (either single copy or duplicated) in the *C. antiqua* gene set (Supplementary Figure S2). This percentage was much larger than those for other gene sets obtained from mRNA-seq analyses of raphidophytes and dinoflagellates causing noxious red tides (Supplementary Table S1) and was comparable or even better those for gene sets obtained from genome sequence analyses of other kinds of algae, protists, and a plant (Supplementary Table S1), supporting the better coverage of the present RNA-seq study than previous ones.

### Comparative Genome and Transcriptome Analysis Among Various Algae

To identify orthologous relationships among the proteins, we performed DomClust orthologous clustering analysis of the 15 proteome sequence sets listed in Supplementary Table S1. We identified 148,421 OGs (including singletons) among which 18,039 contained *C. antiqua* genes, and we examined the presence/absence patterns of orthologs among various algae (Supplementary Figure S3). The number of shared OGs between two genomes is likely to reflect the closeness between them. As expected, *C. subsalsa* and *H. akashiwo* belonging to Raphidophyceae had the largest numbers of shared OGs with *C. antiqua* (Supplementary Figure S4). The next organism that had the largest shared OGs with *C. antiqua* was *Ectocarpus siliculosus*.

We then constructed a phylogenetic tree based on concatenated alignment of universally conserved and nearly one-to-one OGs (Supplementary Figure S5). There were 97 OGs satisfying this condition, yielding a concatenated alignment of length 8487 amino acids. The phylogenetic position of *C. antiqua* was consistent with the above observation from the shared OG analysis.

### Genes Differentially Expressed Between Daytime and Nighttime

We evaluated the expression level of each gene (isoforms of the same gene were merged) in each sample to identify genes that were differentially expressed between daytime and nighttime. As a result, 1,777 and 658 genes were identified as significantly upregulated during the daytime and nighttime, respectively (false discovery rate [FDR] ≤ 0.001). These gene sets were functionally characterized on the basis of GOslim term assignment in terms of biological process (Supplementary Figure S6), cellular component (Supplementary Figure S7), and molecular function (Supplementary Figure S8). We compared four gene sets: all genes, highly expressed genes (the top 1,000 most highly expressed genes in the sample combined with gene sets of daytime and nighttime), genes significantly upregulated during the daytime, and genes significantly upregulated during the nighttime. Some GO terms uniquely appeared in the most frequent categories in either the daytime-specific gene set (highlighted red in Supplementary Figures S6c, S7c, S8c) or nighttime-specific gene set (highlighted blue in Supplementary Figures S6d, S7d, S8d). We also performed Fisher’s exact tests to identify significantly enriched GO terms in either of the differentially expressed gene sets (Supplementary Table S2). GO terms of biological processes enriched in the daytime-specific gene set included “photosynthesis” (GO:0015979; FDR = 2.6 × 10^–6^) and “carbohydrate metabolic process” (GO:0005975; FDR = 2.2 × 10^–9^), and those enriched in the nighttime-specific gene set included “cofactor metabolic process” (GO:0051186; FDR = 4.9 × 10^–4^) and “sulfur compound metabolic process” (GO:0006790; FDR = 7.8 × 10^–3^).

### Genes Related to Photoreception

**FIGURE 1:**
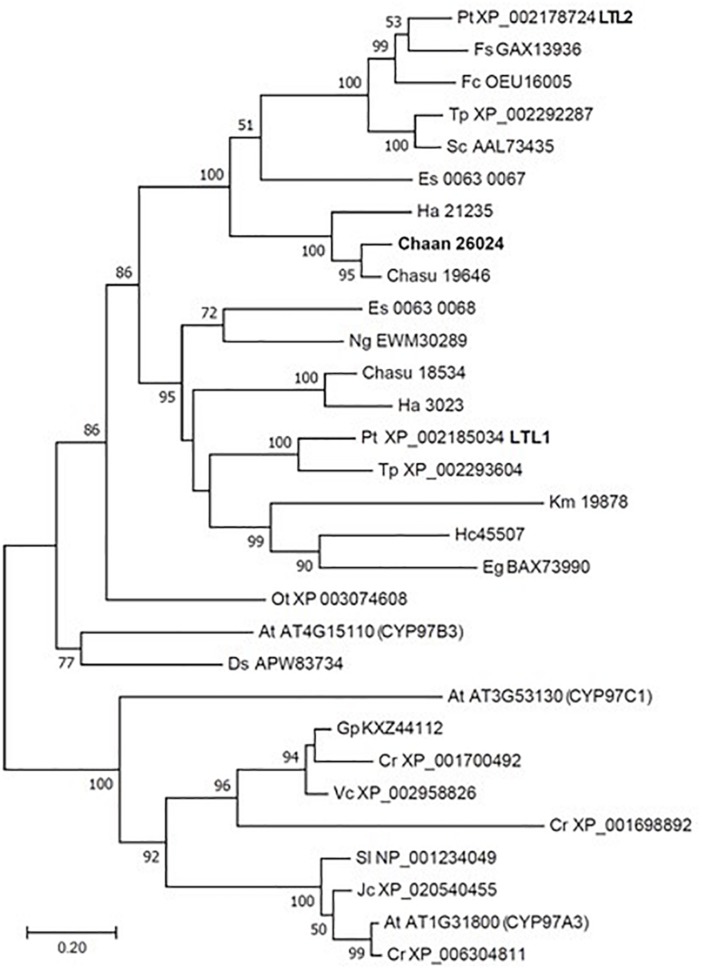
Maximum-likelihood phylogenetic tree of cytochrome P450 monooxygenase (CYP97 type) genes. The tree was inferred using the LG+G model. Numbers represent support values (>50%) obtained with 100 bootstrap replicates. Each gene is labeled with the abbreviated scientific name and accession ID. Abbreviations of scientific names are as follows: (Raphidophyceae, stramenopiles) Chaan, *Chattonella antiqua*; Chasu, *Chattonella subsalsa*; Ha, *Heterosigma akashiwo*; (Bacillariophyceae, stramenopiles); Fs, *Fistulifera solaris*; Fc, *Fragilariopsis cylindrus*; Pt, *Phaeodactylum tricornutum*; Sc, *Skeletonema costatum*; Tp, *Thalassiosira pseudonana*; (Eustigmataceae, stramenopiles) Ng, *Nannochloropsis gaditana*; (Phaeophyceae, stramenopiles) Es, *Ectocarpus siliculosus*; (Brassicaceae) At, *Arabidopsis thaliana*; (Bathycoccaceae) Ot, *Ostreococcus tauri*; (Chlorophyceae) Ds, *Dunaliella salina*; Gp, *Gonium pectoral*; Vc, *Volvox carteri*; Cr, *Chlamydomonas reinhardtii*; (Dinophyceae) Hc, *Heterocapsa circularisquama*; Km, *Karenia mikimotoi*; (Euglenaceae) Eg, *Euglena gracilis*; (Euphorbiaceae) Jc, *Jatropha curcas*; (Solanaceae) Sl, *Solanum lycopersicum*.

Using BLASTP and the RECOG system, we searched for genes related to the photosynthetic pigment pathway and light signal transduction in *C. antiqua* and other red-tide flagellates. First, we searched for genes related to photosynthetic pigment biosynthesis with *Arabidopsis* amino acid sequences as the reference (Castelfranco and Beale, 1983). Almost all genes encoding enzymes required for biosynthesis of chlorophyll *a* were found in *C. antiqua*, as well as the other red-tide flagellates (Supplementary Figure S9); percent identities and *e* -values for *C. antiqua* were 36–67% and ≤2.00E−42, respectively (Supplementary Data S1). Most of the genes encoding enzymes required for biosynthesis of carotenoids were also found in *C. antiqua* and the other red-tide flagellates (Supplementary Figure S10); percent identities and *e* -values for *C. antiqua* were 25–63% and ≤3.00E−12, respectively (Supplementary Data S1). Among genes encoding hydroxylases that convert β-carotene to zeaxanthin, genes for a cytochrome P450 monooxygenase (CYP97 type) was found in *C. antiqua* and the other red-tide flagellates, but those encoding a nonheme di-iron hydroxylase (BCH type) were not found in any red-tide flagellates. Phylogenetic trees of CYP97-type hydroxylase genes are shown in Figure 1 (ML) and Supplementary Figure S11 (NJ, MP). In these trees, the CYP97-type hydroxylase genes seemed to be divided into two groups: algae and plants. In the algae group, the orthologs of the *C. antiqua* CYP97-type hydroxylase gene formed a clade together with the other raphidophytes with strong bootstrap support and were located near clades containing other stramenopiles such as diatoms and brown algae. Genes encoding neoxanthin synthase (NSY) were not found in any red-tide flagellate.

**FIGURE 2:**
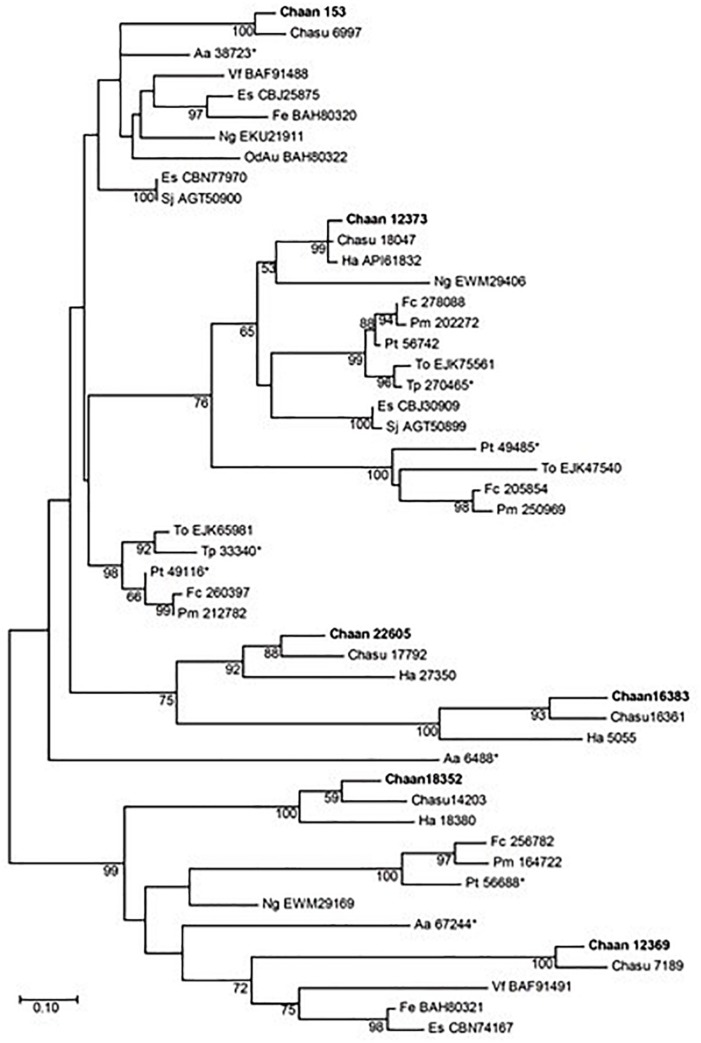
The maximum-likelihood phylogenetic tree of aureochrome genes. The tree was inferred using the Le_Gascuel_2008+gamma model. Numbers represent support values (>50%) obtained with 100 bootstrap replicates. Each gene is labeled with the abbreviated scientific name and accession ID. Sequences with asterisks were obtained from Costa et al. (2013). Abbreviations of scientific names are as follows: (Raphidophyceae) Chaan, *Chattonella antiqua*; Chasu, *Chattonella subsalsa*; Ha, *Heterosigma akashiwo*; (Bacillariophyceae) Fc, *Fragilariopsis cylindrus*; Pt, *Phaeodactylum tricornutum*; Pm, *Pseudo-nitzschia multiseries*; To, *Thalassiosira oceanica*; (Chrysophyceae) Od, *Ochromonas danica*; (Eustigmataceae) Ng, *Nannochloropsis gaditana*; (Pelagomonadaceae) Aa, *Aureococcus anophagefferens*; (Phaeophyceae) Es, *Ectocarpus siliculosus*; Fe, *Fucus evanescens*; Sj, *Saccharina japonica*; (Xanthophyceae) Vf, *Vaucheria frigida*.

Next, we searched for genes encoding photoreceptors, such as blue-absorbing light–oxygen–voltage (LOV) protein, the CRY/PHR family (CPF), and red-absorbing phytochrome (Table 2). By using reference sequences of the xanthophycean algae *Vaucheria frigida*, from which aureochrome was first isolated (Takahashi et al., 2007), orthologous aureochrome genes with a LOV domain and a basic region/leucine zipper (bZIP) domain were identified in raphidophytes only; percent identities and *e* -values for *C. antiqua* were 41–63% and ≤3.00E-52, respectively (Supplementary Data S2). Several genes with LOV domains were found in red-tide dinoflagellates, but no gene for aureochrome was found. Phylogenetic trees of aureochrome genes were constructed using orthologous genes in red-tide flagellates and other stramenopiles (Figure 2 [ML] and Supplementary Figure S12 [NJ, MP]). In the ML tree, four groups were formed, and the aureochrome genes of raphidophytes were divided into each group. Within each group, aureochrome genes of the three raphidophytes formed a clade, with strong bootstrap support.

**TABLE 2:** Photoreceptor genes in raphidophytes and dinoflagellates which form noxious red tides.

		C. antiqua	C. subsalsa	Heterosigma	Heterocapsa	Karenia	**Reference Accession No.**
LOV family							
	Aureochrome	6	6	3	NF	NF	BAF91488
	Others	1	1	3	8	9	AEE78072
Cryptochrome/ photolyase		7	6	5	15	3	AEE28871 and AEE82696
Phytochrome-like		4	2	NF	NF	NF	CBJ33173

*NF, not found.*

**FIGURE 3:**
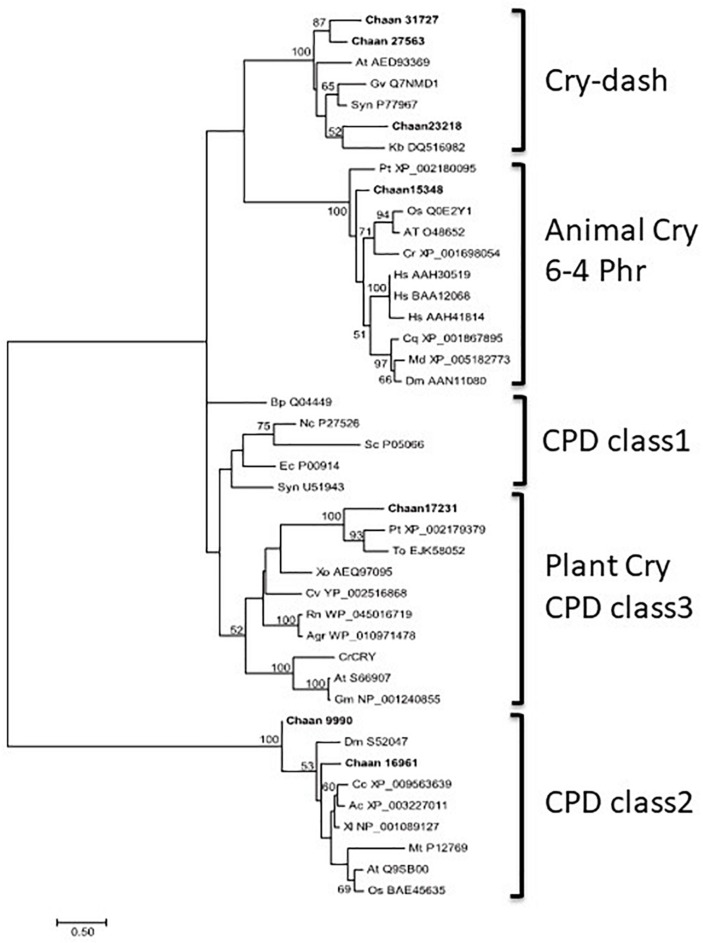
Maximum-likelihood phylogenetic tree of the photolyase/cryptochrome family. The tree was inferred using the LG+G model. Numbers represent support values (>50%) obtained with 100 bootstrap replicates. Each gene is labeled with the abbreviated scientific name and accession ID. Abbreviations of species names are as follows: (raphidophyte) Chaan, *Chattonella antiqua*; (diatom) Pt, *Phaeodactylum tricornutum*; To, *Thalassiosira oceanica*; (dinoflagellate) Kb, *Karenia brevis*; (green algae) Cr, *Chlamydomonas reinhardtii*; (plant) At, *Arabidopsis thaliana*; Gm, *Glycine max*; Os, *Oryza sativa*; (mammal) Hs, *Homo sapiens*; (bird) Cc, *Cuculus canorus*; (amphibia) Xl, *Xenopus laevis*; (reptile) Ac, *Anolis carolinensis*; (insect) Cq, *Culex quinquefasciatus*; Dm, *Drosophila melanogaster*; Md, *Musca domestica*; (fungi) Nc, *Neurospora crassa*; (yeast) Sc, *Saccharomyces cerevisiae*; (cyanobacteria) Gv, *Gloeobacter violaceus*; Syn, *Synechocystis* sp.; (bacteria) Agr, *Agrobacterium tumefaciens*; Bp, *Bacillus pseudofirmus*; Ec, *Escherichia coli*; (proteobacteria) Cv, *Caulobacter vibrioides*; Rn, *Rhizobium nepotum*; Xo, *Xanthomonas oryzae*; (Archaea) Mt, *Methanothermobacter thermautotrophicus*.

Comparison with *Arabidopsis* references revealed orthologous genes of CPF in all five red-tide flagellates examined. In the phylogenic tree, CPF contains four superclasses: 6-4pyrimidine pyrimidone dimer (6-4PHR), the superclass including plant CRY, plant CRY-like, and Class I and III CPDs, CRY-DASH, and the Class II cyclobutane pyrimidine dimer (CPD) photolyases (Fortunato et al., 2015). We constructed phylogenetic trees of CPF genes using orthologous genes in red-tide flagellates and other stramenopiles (Figure 3 and Supplementary Figures S13-1 [ML], S13-2 [NJ, MP]). In these trees, the seven CPF genes of *C. antiqua* seemed to be divided into 6-4PHR, plant CRY or CPD Class III, CPD II, and CRY-DASH (Figure 3); percent identities and *e* -values were 21–50% and ≤1E+00, respectively, in *C. antiqua* and other raphidophytes (Supplementary Data S2). The photolyase homology region (PHR) was found in all CPF orthologous genes of *C. antiqua*. Methylenetetrahydrofolate reductase (MHFR) domain was found in N-terminal site of *Chaan_17231*, which was nested in the superclass including plant CRY, plant CRY-like, and Class I and III CPDs. transcription elongation factor S-II (TFIIS) domain was found in N-terminal site of *Chaan_9990*, which was nested in the CPD Class II.

**FIGURE 4:**
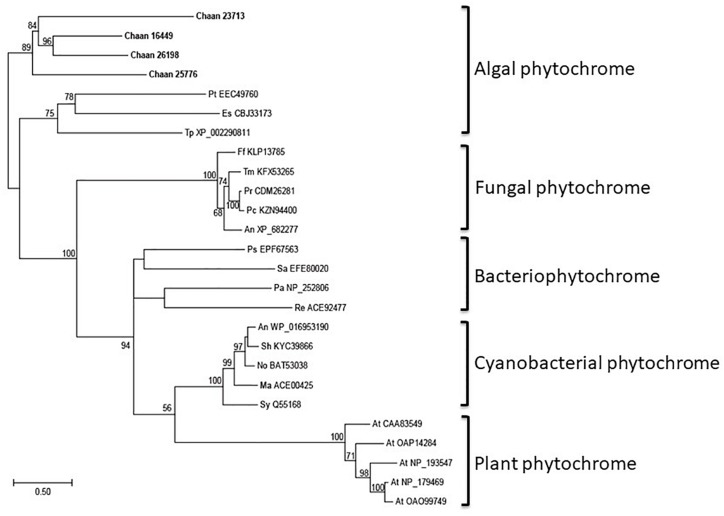
The maximum-likelihood phylogenetic tree of phytochrome family genes. The tree was inferred using the LG+G+I model. Numbers represent support values (>50%) obtained with 100 bootstrap replicates. Each gene is labeled with the abbreviated scientific name and accession ID. Abbreviations of species names are as follows: (raphidophyte) Chaan, *Chattonella antiqua*; (brown algae) Es, *Ectocarpus siliculosus*; (diatom) Pt, *Phaeodactylum tricornutum*; Tp, *Thalassiosira pseudonana*; (plant) At, *Arabidopsis thaliana*; (fungi) An, *Aspergillus nidulans*; Ff, *Fusarium fujikuroi*; Pc, *Penicillium chrysogenum*; Pr, *Penicillium roqueforti*; Tm, *Talaromyces marneffei*; (cyanobacteria) Ma, *Microcystis aeruginosa*; No, *Nostoc* sp.; Sh, *Scytonema hofmannii*; Sy, *Synechocystis* sp.; (bacteria) An, *Anabaena* sp.; Ps, *Pseudomonas syringae*; Sa, *Streptomyces albus*; (proteobacteria) Pa, *Pseudomonas aeruginosa*; Re, *Rhizobium etli*.

Using *Arabidopsis* reference sequences, four orthologous genes of the phytochrome family were identified in *C. antiqua*; percent identities and *e* -values were 23–27% and ≤1.00E−30, respectively (Supplementary Data S2). Using domain search by NCBI, we found no phy-GAF domain, but a domain COG4251 (Bacteriophytochrome: light-regulated signal transduction histidine kinase) which diatom phytochromes with photoreception ability (Fortunato et al., 2016) contain in *Chattonella* phytochromes. Moreover, some cysteine residues were found in the N-terminal photosensory module, indicating that chromophore can bind there. No phytochrome gene was found in other red-tide flagellates than *C. antiqua* and *C. subsalsa*. Phylogenetic trees of phytochrome genes were constructed using orthologous genes in *C. antiqua* and other organisms (Figure 4 [ML] and Supplementary Figure S14 [NJ, MP]). In the ML tree, all phytochrome genes of *C. antiqua* formed a clade located next to the clade of diatom phytochrome genes.

### Genes Related to Nutrient Uptake and Initial Metabolism

**FIGURE 5:**
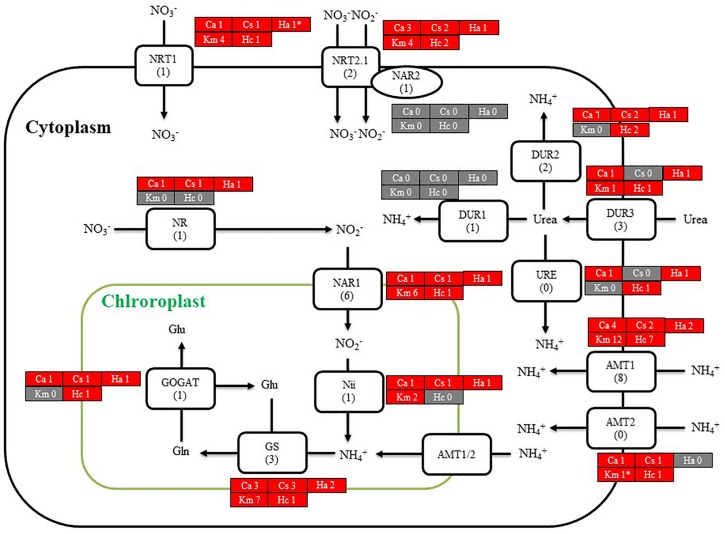
A cell model illustrating the uptake and initial metabolism of nitrogen in *Chattonella antiqua* (Ca), *Chattonella subsalsa* (Cs), *Heterosigma akashiwo* (Ha), *Karenia mikimotoi* (Km), and *Heterocapsa circularisquama* (Hc). Red and gray boxes indicate that orthologous genes were found and not found, respectively, in the RNA-seq data. The numbers next to a species name and below a protein name represent the gene numbers of each species and a model alga, *Chlamydomonasreinhardtii*. Abbreviations of protein names are as follows: AMT, ammonium transporter; DUR, urea transporter; GOGAT, glutamine oxoglutarate amino transferase; GS, glutamine synthetase; NAR, nitrate assimilation-related component; Nii, nitrite reductase; NR, nitrate reductase; NRT, nitrate transporter; and URE, urease.

**FIGURE 6:**
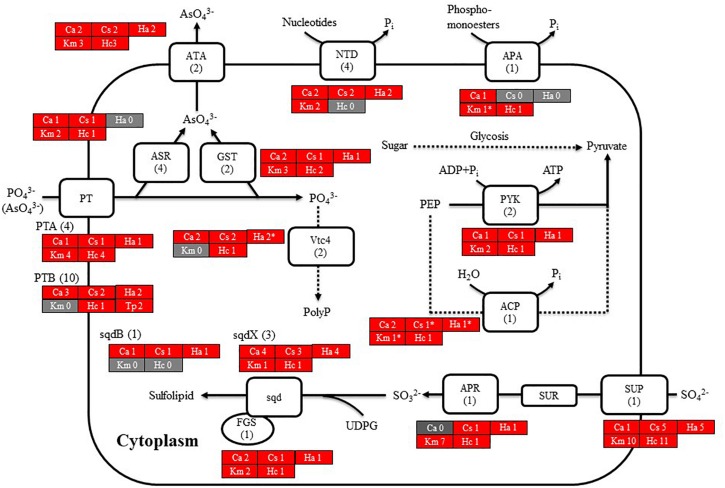
Cell model illustrating the uptake and initial metabolism of phosphate and sulfur compound in *Chattonella antiqua* (Ca), *Chattonella subsalsa* (Cs), *Heterosigma akashiwo* (Ha), *Karenia mikimotoi* (Km), and *Heterocapsa circularisquama* (Hc). Red and gray boxes indicate that orthologous genes were found and not found, respectively, in the RNA-seq data. The numbers next to a species name and below a protein name represent the gene numbers of each species and a model alga, *Chlamydomonasreinhardtii*. Abbreviations of protein names are as follows: ACP, acid phosphatase; APA, alkaline phosphatase; APR, adenosine-5′-phosphosulfate reductase; ASR, arsenate reductase; ATA, arsenite translocating ATPase; FGS, ferredoxin-dependent glutamate synthase; GST, glutathione S-transferase; NTD, 5′-nucleotidase; sqd, sulfolipid SQDG biosynthesis protein; PT, proton/phosphate symporter; PYK, pyruvate kinase; and Vtc4, vacuolar transporter chaperone 4.

**FIGURE 7:**
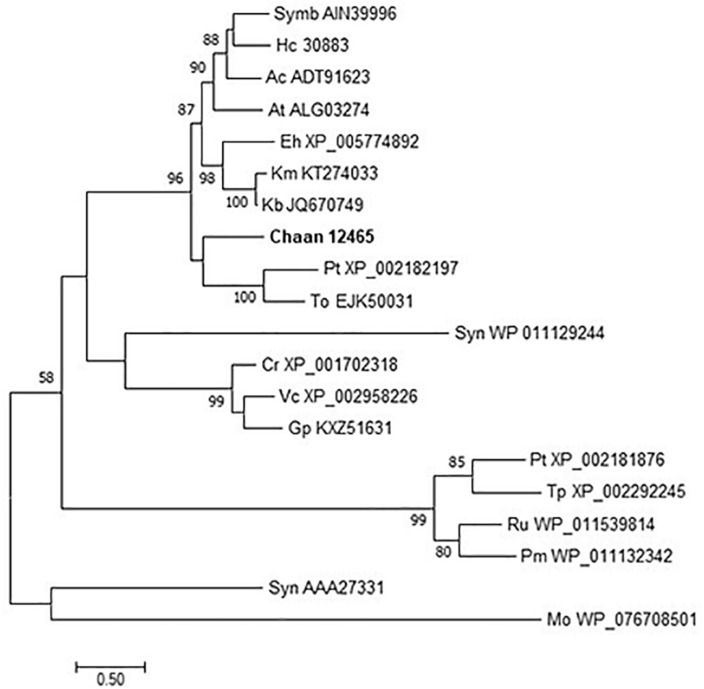
The maximum-likelihood phylogenetic tree of alkaline phosphatase genes. The tree was inferred using the WAG+G model. Numbers represent support values (>50%) obtained with 100 bootstrap replicates. Each gene is labeled with the abbreviated scientific name and accession ID. Abbreviations of species names are as follows: (Raphidophyceae) Chaan, *Chattonella antiqua*; (Bacillariophyceae) Pt, *Phaeodactylum tricornutum*; To, *Thalassiosira oceanica*; Tp, *Thalassiosira pseudonana*; (Chlorophyceae) Cr, *Chlamydomonas reinhardtii*; Gp, *Gonium pectoral*; Vc, *Volvox carteri*; (Dinophyceae) At, *Alexandrium tamarense*; Ac, *Amphidinium carterae*; Hc, *Heterocapsa circularisquama*; Kb, *Karenia brevis*; Km, *Karenia mikimotoi*; Symb, *Symbiodinium* sp.; (Prymnesiophyceae) Eh, *Emiliania huxleyi*; (cyanobacteria) Syn, *Synechocystis* sp.; Pm, *Prochlorococcus marinus*; Syn, *Synechococcus* sp.; (bacteria) Mo, *Microbacterium oleivorans*; and (proteobacteria) Ru, *Ruegeria* sp.

We then searched for genes related to the uptake of and initial steps in the metabolism of nitrogen and phosphorus in *C. antiqua* and the other red-tide flagellates by using reference sequences of a model microalga, *Chlamydomonas reinhardtii* (chlorophyte) (Sanz-Luque et al., 2015; Dyhrman, 2016; Supplementary Data S3). As observed for the other red-tide flagellates, orthologous genes encoding most of the enzymes required for uptake and initial metabolism of nitrogen and phosphorus were found in *C. antiqua* (Figures 5, 6); percent identities and *e* -values were 28–57% and ≤9E-13 for nitrogen-related genes and 24–54% and ≤9E-10 for phosphorus-related genes (Supplementary Data S3). Orthologous genes of alkaline phosphatase (APA), which is required for utilization of organic phosphorus, were found in *C. antiqua*, *K. mikimotoi*, and *H. circularisquama*. Multi-sequence alignment showed that four motifs (Lin et al., 2015) are conserved in the APA genes of these red-tide flagellates (Supplementary Figure S15). Phylogenetic trees of APA genes were constructed using orthologous genes in *C. antiqua* and other red-tide flagellates (Figure 7 [ML] and Supplementary Figure S16 [NJ and MP]). In the phylogenetic trees, the APA genes of red-tide flagellates formed a clade with the same or a closely related taxonomic group: for instance, the APA gene of *C. antiqua* was nested in the same clade as APA genes of diatoms.

### Genes Related to Ichthyotoxicity

**FIGURE 8:**
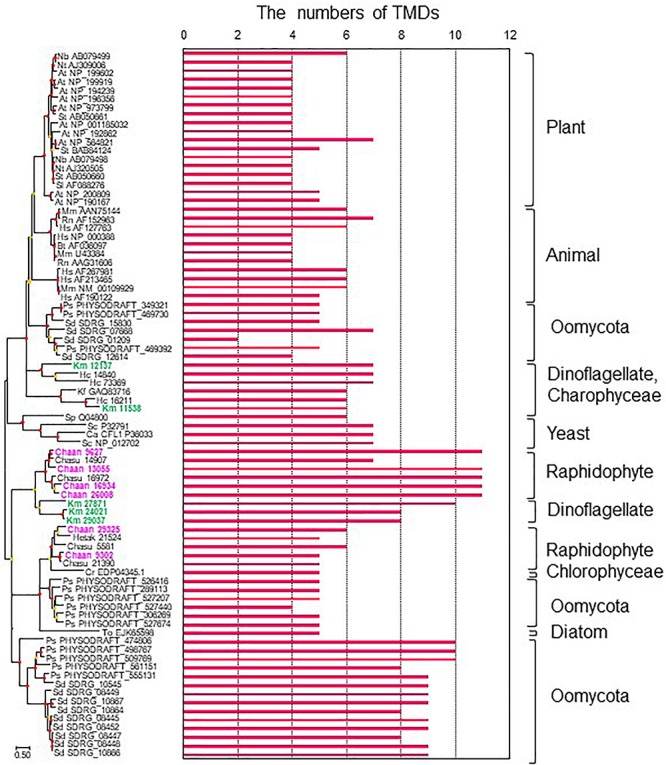
The maximum-likelihood phylogenetic tree and the numbers of transmembrane domains (TMDs) of NADPH oxidase genes. The tree was inferred using the LG+G+I model. Red and orange circles on nodes represent that the bootstrap value was ≥80% and 50–79%, respectively. Each gene is labeled with the abbreviated scientific name and accession ID. Abbreviations of species names are as follows: (Raphidophyceae) Chaan, *Chattonella antiqua*; Chasu, *Chattonella subsalsa*; Ha, *Heterosigma akashiwo*; (Bacillariophyceae) To, *Thalassiosira oceanica*; (Chlorophyceae) Cr, *Chlamydomonas reinhardtii*; (Dinophyceae) Km, *Karenia mikimotoi*; Hc, *Heterocapsa circularisquama*; (Klebsormidiophyceae) Kf, *Klebsormidium flaccidum*; (Oomycota) Ps, *Phytophthora sojae*; Sd, *Saprolegnia diclina*; (plant) At, *Arabidopsis thaliana*; Nb, *Nicotiana benthamiana*; Nt, *Nicotiana tabacum*; Sl, *Solanum lycopersicum*; St, *Solanum tuberosum*; (mammal) Bt, *Bos Taurus*; Hs, *Homo sapiens*; Mm, *Mus musculus*; Rn, *Rattus norvegicus*; (yeast) Sp, *Schizosaccharomyces pombe*; Sc, *Saccharomyces cerevisiae*; and (imperfect yeast) Ca, *Candida albicans*. The numbers of TMDs were predicted by TMHMM Server v. 2.0.

To identify NOX genes in *C. antiqua* and the other red-tide flagellates we conducted searches using the sequence of *Arabidopsis* respiratory burst oxidase homolog protein A gene as the reference (Supplementary Data S3). Six NOX genes were found in *C. antiqua*; percent identities and *e* -values were 22–29% and ≤1.00E−14, respectively. In each of the other red-tide flagellates, two to four NOX genes were found. Multi-sequence alignment showed that four motifs, i.e., FAD-isoalloxazine binding site, motif 2, NADPH-ribulose binding site, and NADPH binding site (Finegold et al., 1996; Torres et al., 1998; Hervé et al., 2006) were conserved with high identities in the NOX genes of *C. antiqua* (Supplementary Figure S17). However, an EF-hand domain was not found in any NOX genes in the red-tide flagellates. Analyses using the TMHMM Server v. 2.0 to predict transmembrane helices in proteins indicated that two of the six *C. antiqua* NOX genes had five or six transmembrane domains (TMDs) (*Chaan 29325, 9302*), and the others had 11 TMDs (*Chaan 9627, 13055, 16934, 26008*) (Figure 8 and Supplementary Figure S18). These two types of *C. antiqua* NOX genes fell into different clades in the ML phylogenic tree (Figure 8). Similarly, some NOX genes of the other red-tide flagellates, i.e., *C. subsalsa* and *K. mikimotoi*, contained several TMDs (*Chasu 21390, 5581, Km 24021, 27871, 29037*) and others contained more TMDs (*Chasu 14907, 16972, Km 12137, 11538*), which were phylogenetically divided into clades in the same manner as the *C. antiqua* genes. All NOX genes of *H. circularisquama* were the type with several TMDs.

## Discussion

In the present study, we conducted RNA-seq of *Chattonella antiqua* to identify genes with functions closely related to the dynamics of a noxious red tide. The read counts (371 million reads) were higher than those in previous studies of red-tide flagellates (10–242 million reads; Table 1). As a result, the contig number (84,309–191,212 contigs) was lower, and N50 and mean length of the contigs were slightly larger than those in the previous studies, with the exception of *Karenia brevis* (N50, ∼1,500 nt; mean length, ∼1,000, Ryan et al., 2014). This taken together with the results of the BUSCO analysis, where more than 90% of the eukaryotic BUSCOs were identified as complete in the *C. antiqua* gene set (Supplementary Figure S2), suggests that the larger number of reads used in the present study allowed us to obtain improved assembly of mRNA sequences of *C. antiqua*. However, by searching the NR database, we were able to assign functional annotations to only a small fraction of contigs (<40%). This was in part because no CDS was predicted in many contigs, suggesting the existence of non-coding RNAs, and in part because information on the genomes and functions of raphidophytes is poor.

In GO analysis, transcripts assigned to terms such as “photosynthesis” and “carbohydrate metabolic process” were significantly enriched in the genes expressed predominantly in the daytime (Supplementary Table S2). This was as expected because cellular carbon content in *C. antiqua* increases due to photosynthesis during the light period (Kohata and Watanabe, 1988). GO terms such as “DNA metabolic process” and “chromosome organization” uniquely appeared in the most frequent categories in the nighttime-specific gene set, although these terms were not significantly enriched (Supplementary Figure S6). Again, this was as expected because the nuclear DNA content of *C. antiqua* (G1/S transition) begins to increase ∼10 h after the onset of light irradiation, and cell division occurs during the nighttime (Nemoto and Furuya, 1985; Nemoto et al., 1987). The gene ontology data thus indicate that the sequence data roughly covered representative transcripts expressed during daytime and nighttime.

Using the reference sequences of model organisms and the RECOG system, orthologous genes related to processes important for red-tide development and toxicity—photosynthesis, photoreception, nutrient uptake, and superoxide production—were identified in the RNA-seq data of *C. antiqua*. As in other harmful red-tide species, orthologous genes of almost all enzymes required for biosynthesis of chlorophyll *a* and carotenoids were found in *C. antiqua* (Supplementary Figures S9, S10). In the carotenoid biosynthesis system, the NSY gene was not found in *C. antiqua* or other red-tide flagellates; this was as predicted because neoxanthin has not been detected in raphidophytes or dinoflagellates (Takaichi, 2011). Hydroxylation is required for the biosynthesis of xanthophylls from carotenes (Tian and DellaPenna, 2004). In plants, there are two types of carotenoid hydroxylase genes, i.e., CYP97 type and BCH type, which are reported to play a role in hydroxylation of α-carotene and β-carotene, respectively (Ruiz-Sola and Rodríguez-Concepción, 2012). Although raphidophytes and dinoflagellates do not produce α-carotene (Takaichi, 2011), only CYP97-type genes were found in the five red-tide algae. According to the phylogenetic tree (Figure 1), the *C. antiqua* CYP97-type gene belongs to the same clade as genes of lutein deficient like proteins (LUT) in diatoms (LTL1, LTL2), which are putative genes encoding CYP97-type enzymes involved in the hydroxylation of β-carotene (Coesel et al., 2008; Bertrand, 2010). Similarly, the CYP97-type genes in the other five red-tide species also belonged to the same clades as LTL1 and LTL2. These findings indicate that coastal phytoplankters such as raphidophytes, diatoms and dinoflagellates may universally utilize CYP97-type genes for hydroxylation of β-carotene.

Recently, a plethora of photoreceptor-like sequences from marine microalgae have been identified by omics approaches, and their functions and structures have been thoroughly analyzed (Jaubert et al., 2017). Some swimming behaviors, such as diurnal vertical migration and negative phototaxis, are regulated by specific spectral bands of light in *C. antiqua* and other red-tide flagellates (Shikata et al., 2013, 2015, 2016). The present study identified genes for blue light receptors such as aureochrome and CRY and a red/far-red light receptor phytochrome. In addition to the aureochrome gene reported previously (Ishikawa et al., 2009), the present study newly identified five aureochrome genes in *C. antiqua* (Figure 2). In *H. akashiwo*, one of the four aureochrome genes (*Haaureo1*) exhibits a clear diel rhythm, with the highest and the lowest transcript abundances occurring at dawn and dusk under a light:dark cycle, although the rhythm disappears under continuous dark conditions (Ji et al., 2017). Here, one of the aureochrome genes in *C. antiqua* (*Chaan22605*) was shown to belong to the same clade as *Haaureo1*. We cannot strictly compare the expression patterns between *Chaan22605* and *Haaureo1* because the light:dark cycle used by Ji et al. (2017) differed from that used here. Nevertheless, we observed that the expression level of *Chaan22605* was significantly higher in the daytime (roughly corresponding to T4 in Ji et al., 2017) than in the nighttime (corresponding to T12 in Ji et al., 2017) (Supplementary Data S2), which corresponds with the expression rhythm of *Haaureo1*.

CPF contains photolyase and CRY. Under blue light irradiation, the photolyase repairs DNA damage caused by ultraviolet exposure. The photolyase has two types, i.e., CPD and 6-4PHR (Lin and Todo, 2005), of which CPD is phylogenetically divided into Class I (in prokaryotes) and Class II (in eukaryotes). CRY contributes to photomorphogenesis as a blue light receptor in plants and controls circadian rhythm in animals. According to sequence similarities and phylogenetical analyses, CRYs are clustered into three subfamilies: plant CRY, animal CRY, and CRY-DASH. CPF genes have also been reported in diatoms and dinoflagellates and are suspected of contributing to light-dependent regulation of the cell cycle and circadian rhythm (Brunelle et al., 2007; Oliveri et al., 2014). Here, *C. antiqua* genes encoding 6-4PHR, plant CRY or CPD Class III, CPD Class II, and CRY-DASH were identified (Figure 3).

All phytochrome genes of *C. antiqua* seem to be nested within a clade of algal phytochrome genes (Figure 4). As in plants, physiological phenomena of microalgae are regulated by red light. Precision of geotaxis in the green alga *C. reinhardtii*, and alterations of cell speed during phototaxis of benthic diatoms, are regulated by red light (Sineshchekov et al., 2000; McLachlan et al., 2009). It is suspected that phytochromes may contribute to these physiological phenomena, because *C. reinhardtii* and diatoms have phytochrome genes (Bowler et al., 2008). In *C. antiqua*, the negative geotaxis during the daytime disappears under red light, regardless of irradiation direction (Shikata et al., 2016). These findings suggest that photoreceptors, such as aureochrome, CPF, and phytochrome, may be involved in the regulation of swimming behaviors in *C. antiqua*; however, biochemical and functional analyses such as transformation techniques and dsRNA transfection (RNAi) are required to verify the contribution of photoreceptors to each swimming trait.

*Chattonella antiqua* can utilize dissolved inorganic nitrogen (nitrate, nitrite, and ammonium) and urea as a nitrogen source for growth (Nakamura and Watanabe, 1983; Fukao et al., 2007), and dissolved inorganic phosphorus as a phosphorus source (Nakamura and Watanabe, 1983). The present study revealed orthologous genes of transporters and enzymes required for uptake and primary metabolism of nitrogen and phosphorus (Figures 5, 6). Orthologs of transporters for uptakes of sulfate ion and enzymes for synthesis of sulfolipid (sulfoquinovosyldiacylglycerol) which act as a substitute for phospholipids under phosphorus deficient conditions (Dyhrman, 2016) were also identified (Figure 6).

Nakamura (1985) reported that *C. antiqua* cannot utilize dissolved organic phosphorus and has no APA activity; however, an ortholog gene of APA was found in the present study. It has been reported that *C. marina* and *C. ovata* can grow not only with inorganic phosphorus but also with some kinds of dissolved organic phosphorus (Yamaguchi et al., 2008; Wang et al., 2011). Opinions about the ability to utilize dissolved organic phosphorus and to produce APA in *H. akashiwo* differ (Yamaguchi et al., 2004; Wang et al., 2011; Wang and Liang, 2015), but no ortholog gene of APA was detected in the RNA-seq data of *H. akashiwo* in the present study. On the other hand, Haley et al. (2017) reported that expression level of the APA gene was significantly increased under low phosphorus conditions in *H. akashiwo*. Differences in culture conditions may change a level of the gene expression of APA, and the ability to produce APA may be different among each culture strains.

To our knowledge, the present study is the first report on putative sequences of NOX genes of raphidophytes, including *C. antiqua*, although a previous report of a Southern blot analysis has suggested the presence of a gene encoding a gp91phox homolog in *C. marina* (Kim et al., 2000). Moreover, we also found multiple NOX genes in the other four red-tide flagellates examined. We observed that none of the NOX genes of the five red-tide flagellates had an EF-hand domain; absence of such a domain appears to be a common feature in algal NOX genes (Anderson et al., 2011). Ca^2+^ signaling through the EF-hand domain contributes to regulation of ROS production in plants (Sagi and Fluhr, 2006; Kadota et al., 2015), but algae may have another mechanism. Two types of NOX genes were found in *C. antiqua* : one with several TMDs and another with numerous TMDs (Figure 8). Interestingly, the red-tide dinoflagellate *K. mikimotoi* (Alveolata), which is not phylogenically close to *C. antiqua* (Stramenopiles) but kills fishes and produces relatively large amounts of ROS in the similar way (Yamasaki et al., 2004), also has NOX genes with numerous TMDs; these genes belong to the clade of *C. antiqua* NOX genes with numerous TMDs in the phylogenetic tree (Figure 8). Most animals and plants have multiple NOX genes with different induction patterns and functions (Canton and Grinstein, 2014). It is reported that the superoxide production level is higher during the light period than during the dark period in *C. antiqua* (Kim et al., 2005). Two genes of NOX with numerous TMDs (*Chaan26008*, *Chaan16934*) expressed significantly higher during the daytime than during the night time (Supplementary Data S4, FDR > 0.001). *Chattonella* cells stimulated with fish mucus and galacturonic acid increased the generation of superoxide (Nakamura et al., 1998; Kim et al., 2000). It is interesting how two types of *Chattonella* NOX work to produce superoxide.

In the present study, RNA-seq and comparative analyses with other organisms identified genes related to biosynthesis of pigments for photosynthesis, light signal transduction, nutrient uptake, and ichthyotoxicity of *C. antiqua*. The next step is functional analysis of each gene to enrich our understanding of the eco-physiological features of marine raphidophytes at the molecular level.

## Data Availability

The datasets generated for this study can be found in DDBJ, BioProject IDs PRJDB7469 and PRJDB7513.

## Author Contributions

TS designed the research projects, carried out the sampling from algal cultures, performed the phylogenic analyses, and wrote the manuscript. FT performed the phylogenic analyses and wrote a part of the manuscript. HN performed the bioinformatic analyses. ShS sequenced the mRNA of *C. antiqua*. YK designed the project and promoted acquirement of the research grants. SeS prepared the algal culture samples. KY and YN provided the information on photosynthesis and wrote a part of the section “Discussion.” YY provided the information on toxicity of red-tide flagellates and wrote a part of the section “Discussion.” IU performed the bioinformatic analyses and wrote a part of the manuscript. All authors read and approved the final manuscript.

## Conflict of Interest Statement

The authors declare that the research was conducted in the absence of any commercial or financial relationships that could be construed as a potential conflict of interest.

## Footnotes

1 https://github.com/TransDecoder/TransDecoder/wiki

2 http://mbgd.genome.ad.jp/RECOG/
