# Supplementary material for: RNA-Seq Analysis Reveals Genes Related to Photoreception, Nutrient Uptake, and Toxicity in a Noxious Red-Tide Raphidophyte Chattonella antiqua
Source: Front Microbiol. 2019 Jul 31;10:1764. doi: 10.3389/fmicb.2019.01764 (PMC6685483; doi:10.3389/fmicb.2019.01764)
Supplement: Supplementary file 1 [file Table_1.DOCX]

**Table S1 Data sources used for comparative analysis.**

| Species | Type^2^ | Source | Accession^3^ |
| --- | --- | --- | --- |
| Heterokonta |  |  |  |
| Raphidophyceae |  |  |  |
| *Chattonella antiqua* | T | this work |  |
| *Chattonella subsalsa* | T | SRA | SRR1300240 |
| *Heterosigma akashiwo* | T | SRA | SRR1296916 |
| Phaeophyceae |  |  |  |
| *Ectocarpus siliculosus* | G | GenBank | PRJEA42625 |
| Bacillariophyta |  |  |  |
| *Phaeodactylum tricornutum* | G | RefSeq | PRJNA33251 |
| Mediophyceae |  |  |  |
| *Thalassiosira pseudonana* | G | RefSeq | PRJNA34119 |
| Oomycota |  |  |  |
| *Saprolegnia diclina* | G | RefSeq | PRJNA255245 |
| *Phytophthora sojae* | G | RefSeq | PRJNA262907 |
| Eustigmatophyceae |  |  |  |
| *Nannochloropsis gaditana* | G | RefSeq | PRJNA224372 |
| Haptophyta |  |  |  |
| Prymnesiophyceae |  |  |  |
| *Emiliania huxleyi* | G | RefSeq | PRJNA222302 |
| Alveolata |  |  |  |
| Dinophyceae |  |  |  |
| *Karenia mikimotoi* | T | Kimura et al. 2015 |  |
| *Heterocapsa circularisquama* | T | this work |  |
| Archaeplastida |  |  |  |
| Cyanidiophyceae |  |  |  |
| *Cyanidioschyzon merolae* | G | GenBank | PRJNA10792 |
| Viridiplantae |  |  |  |
| Chlorophyceae |  |  |  |
| *Chlamydomonas reinhardtii* | G | RefSeq | PRJNA21061 |
| Angiosperms |  |  |  |
| *Arabidopsis thaliana* | G | RefSeq | PRJNA116 |

^1^ Classification according to the NCBI Taxonomy database.

^2^ Data source type is either transcriptome (T) or genome (G).

^3^ BioProject ID for genomic data or SRA ID for transcriptome data.

| **Table S2** GO enrichment analysis based on Fisher's exact test | |  |  |  |
| --- | --- | --- | --- | --- |
| (A) Genes upregulated during daytime | |  |  |  |
| GO-ID | Term | Category | FDR | P-Value |
| GO:0009536 | plastid | C | 1.41E-09 | 4.69E-12 |
| GO:0005975 | carbohydrate metabolic process | P | 2.17E-09 | 1.56E-11 |
| GO:0009579 | thylakoid | C | 2.17E-09 | 2.17E-11 |
| GO:0006091 | generation of precursor metabolites and energy | P | 4.32E-09 | 5.76E-11 |
| GO:0015979 | photosynthesis | P | 2.63E-06 | 4.39E-08 |
| GO:0016491 | oxidoreductase activity | F | 5.26E-06 | 1.05E-07 |
| GO:0003824 | catalytic activity | F | 2.09E-05 | 4.87E-07 |
| GO:0044710 | single-organism metabolic process | P | 6.68E-04 | 1.78E-05 |
| GO:0051186 | cofactor metabolic process | P | 2.96E-03 | 8.87E-05 |
| GO:0016301 | kinase activity | F | 6.74E-03 | 2.25E-04 |
| GO:0044281 | small molecule metabolic process | P | 8.82E-03 | 3.23E-04 |
| GO:0016772 | transferase activity, transferring phosphorus-containing groups | F | 2.07E-02 | 8.26E-04 |
|  |  |  |  |  |
| (B) Genes upregulated during nighttime | |  |  |  |
| GO-ID | Term | Category | FDR | P-Value |
| GO:0044281 | small molecule metabolic process | P | 6.84E-11 | 2.28E-13 |
| GO:0044710 | single-organism metabolic process | P | 3.69E-10 | 2.46E-12 |
| GO:0006082 | organic acid metabolic process | P | 3.98E-06 | 7.96E-08 |
| GO:0006520 | cellular amino acid metabolic process | P | 3.98E-06 | 7.96E-08 |
| GO:0043436 | oxoacid metabolic process | P | 3.98E-06 | 7.96E-08 |
| GO:0019752 | carboxylic acid metabolic process | P | 3.98E-06 | 7.96E-08 |
| GO:0044699 | single-organism process | P | 7.07E-05 | 1.65E-06 |
| GO:1901564 | organonitrogen compound metabolic process | P | 2.40E-04 | 6.41E-06 |
| GO:0009058 | biosynthetic process | P | 4.90E-04 | 1.58E-05 |
| GO:0044237 | cellular metabolic process | P | 4.90E-04 | 1.76E-05 |
| GO:0051186 | cofactor metabolic process | P | 4.90E-04 | 1.80E-05 |
| GO:0006807 | nitrogen compound metabolic process | P | 5.92E-04 | 2.37E-05 |
| GO:0006091 | generation of precursor metabolites and energy | P | 1.15E-03 | 4.97E-05 |
| GO:0009987 | cellular process | P | 1.44E-03 | 6.73E-05 |
| GO:0003824 | catalytic activity | F | 1.54E-03 | 7.72E-05 |
| GO:0016491 | oxidoreductase activity | F | 3.69E-03 | 1.97E-04 |
| GO:0043167 | ion binding | F | 5.26E-03 | 3.00E-04 |
| GO:0016765 | transferase activity, transferring alkyl or aryl (other than methyl) groups | F | 5.26E-03 | 3.17E-04 |
| GO:0008152 | metabolic process | P | 5.26E-03 | 3.33E-04 |
| GO:0005694 | chromosome | C | 5.89E-03 | 3.93E-04 |
| GO:0006790 | sulfur compound metabolic process | P | 7.80E-03 | 5.46E-04 |
| GO:0005488 | binding | F | 9.64E-03 | 7.07E-04 |
| GO:0015979 | photosynthesis | P | 1.57E-02 | 1.20E-03 |
| GO:0016740 | transferase activity | F | 2.67E-02 | 2.13E-03 |
| GO:0016301 | kinase activity | F | 4.33E-02 | 3.61E-03 |
