# Supplementary material for: RNA-Seq Analysis Reveals Genes Related to Photoreception, Nutrient Uptake, and Toxicity in a Noxious Red-Tide Raphidophyte Chattonella antiqua
Source: Front Microbiol. 2019 Jul 31;10:1764. doi: 10.3389/fmicb.2019.01764 (PMC6685483; doi:10.3389/fmicb.2019.01764)
Supplement: Supplementary file 6 [file Presentation_1.pptx]

## Slide 1
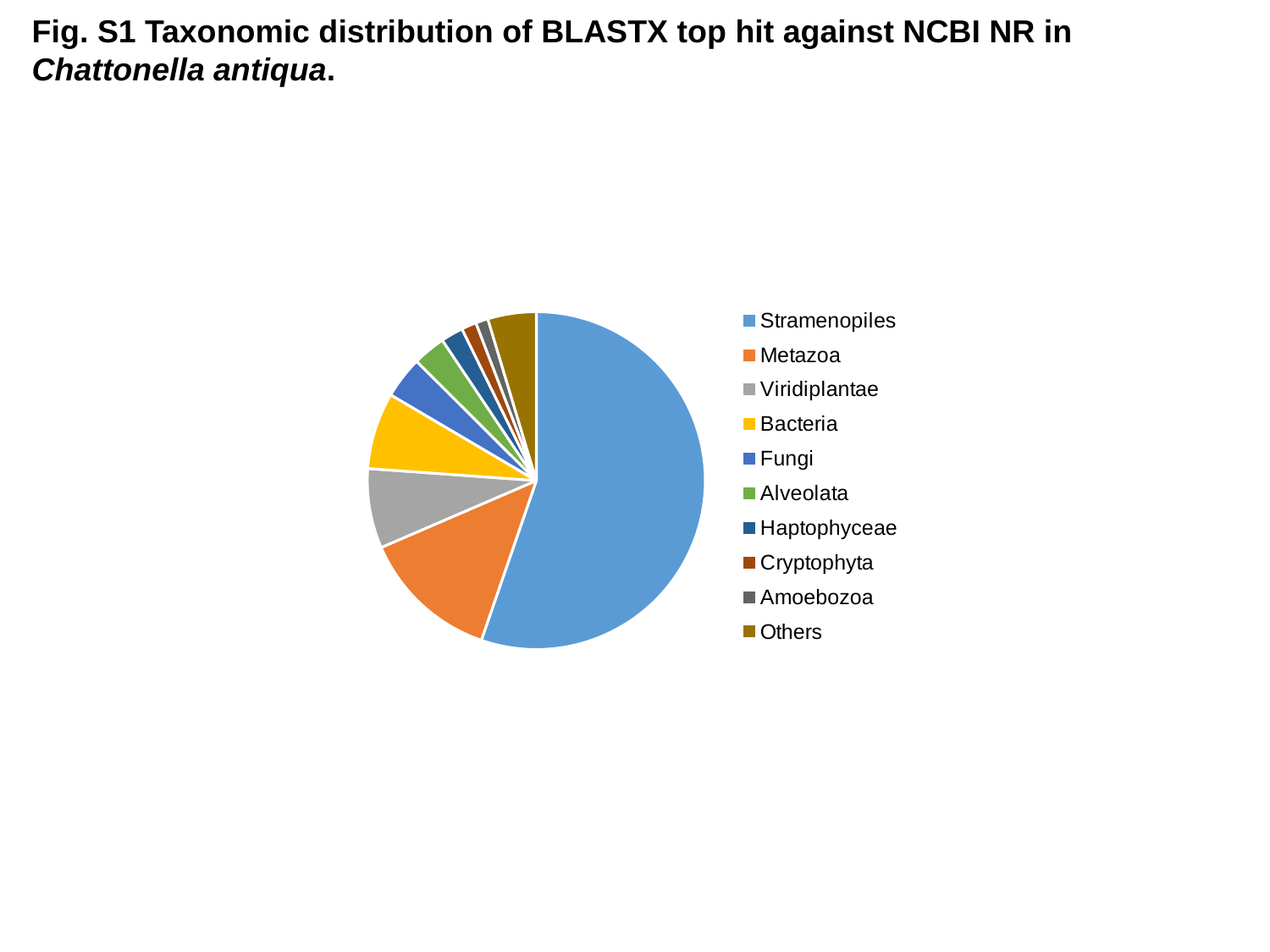

Fig. S1 Taxonomic distribution of BLASTX top hit against NCBI NR in Chattonella antiqua.
### Chart
| Category | |
|---|---|
| Stramenopiles | 14164.0 |
| Metazoa | 3391.0 |
| Viridiplantae | 1955.0 |
| Bacteria | 1880.0 |
| Fungi | 1025.0 |
| Alveolata | 804.0 |
| Haptophyceae | 546.0 |
| Cryptophyta | 367.0 |
| Amoebozoa | 303.0 |
| Others | 1188.0 |

## Slide 2
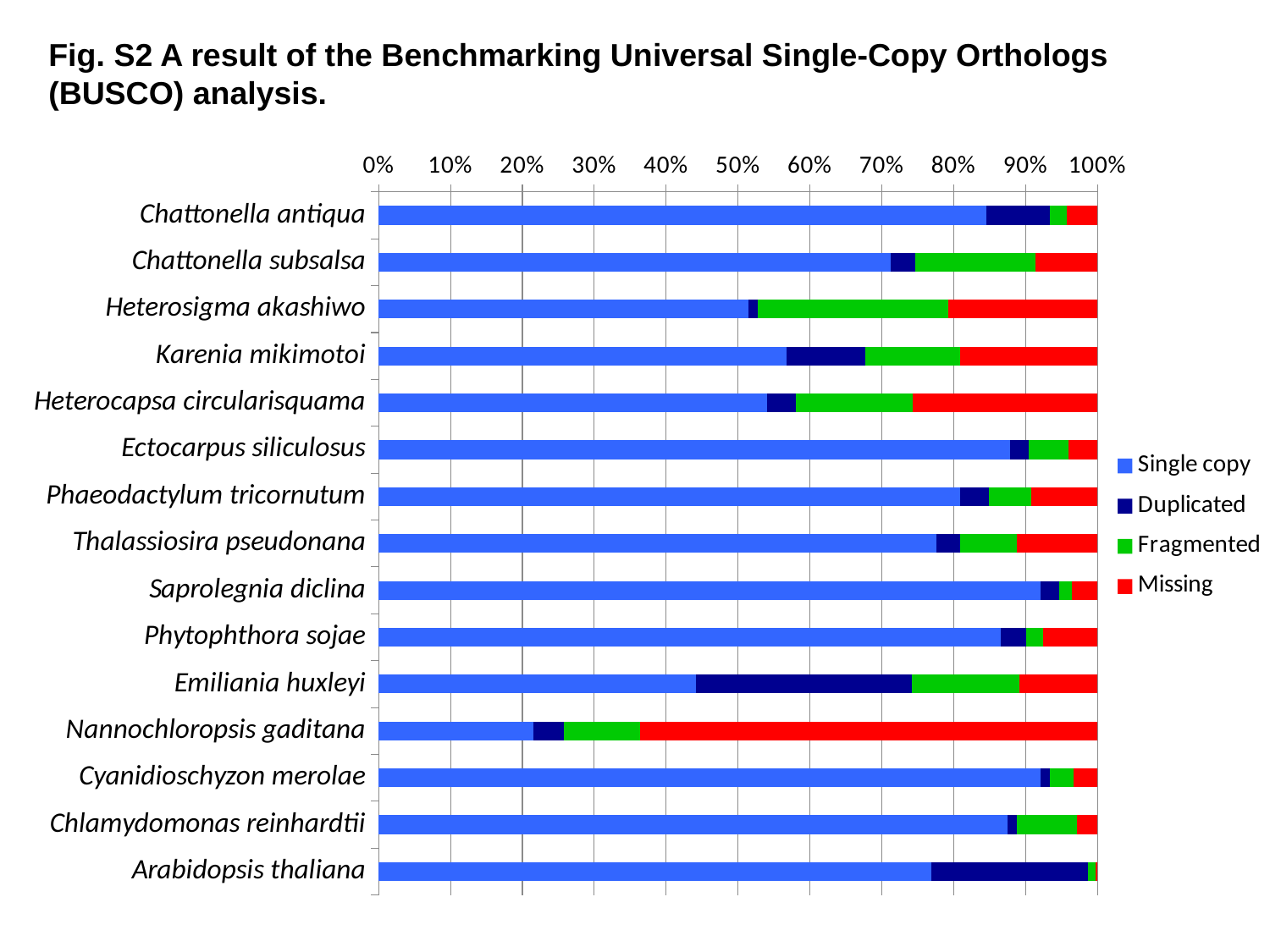

Fig. S2 A result of the Benchmarking Universal Single-Copy Orthologs (BUSCO) analysis.
### Chart
| Category | Single copy | Duplicated | Fragmented | Missing |
|---|---|---|---|---|
| Chattonella antiqua | 84.5 | 8.9 | 2.3 | 4.3 |
| Chattonella subsalsa | 71.3 | 3.3 | 16.8 | 8.6 |
| Heterosigma akashiwo | 51.5 | 1.3 | 26.4 | 20.8 |
| Karenia mikimotoi | 56.8 | 10.9 | 13.2 | 19.1 |
| Heterocapsa circularisquama | 54.1 | 4.0 | 16.2 | 25.7 |
| Ectocarpus siliculosus | 87.8 | 2.6 | 5.6 | 4.0 |
| Phaeodactylum tricornutum | 80.9 | 4.0 | 5.9 | 9.2 |
| Thalassiosira pseudonana | 77.6 | 3.3 | 7.9 | 11.2 |
| Saprolegnia diclina | 92.1 | 2.6 | 1.7 | 3.6 |
| Phytophthora sojae | 86.5 | 3.6 | 2.3 | 7.6 |
| Emiliania huxleyi | 44.2 | 30.0 | 14.9 | 10.9 |
| Nannochloropsis gaditana | 21.5 | 4.3 | 10.6 | 63.6 |
| Cyanidioschyzon merolae | 92.1 | 1.3 | 3.3 | 3.3 |
| Chlamydomonas reinhardtii | 87.5 | 1.3 | 8.3 | 2.9 |
| Arabidopsis thaliana | 76.9 | 21.8 | 1.0 | 0.3 |

## Slide 3
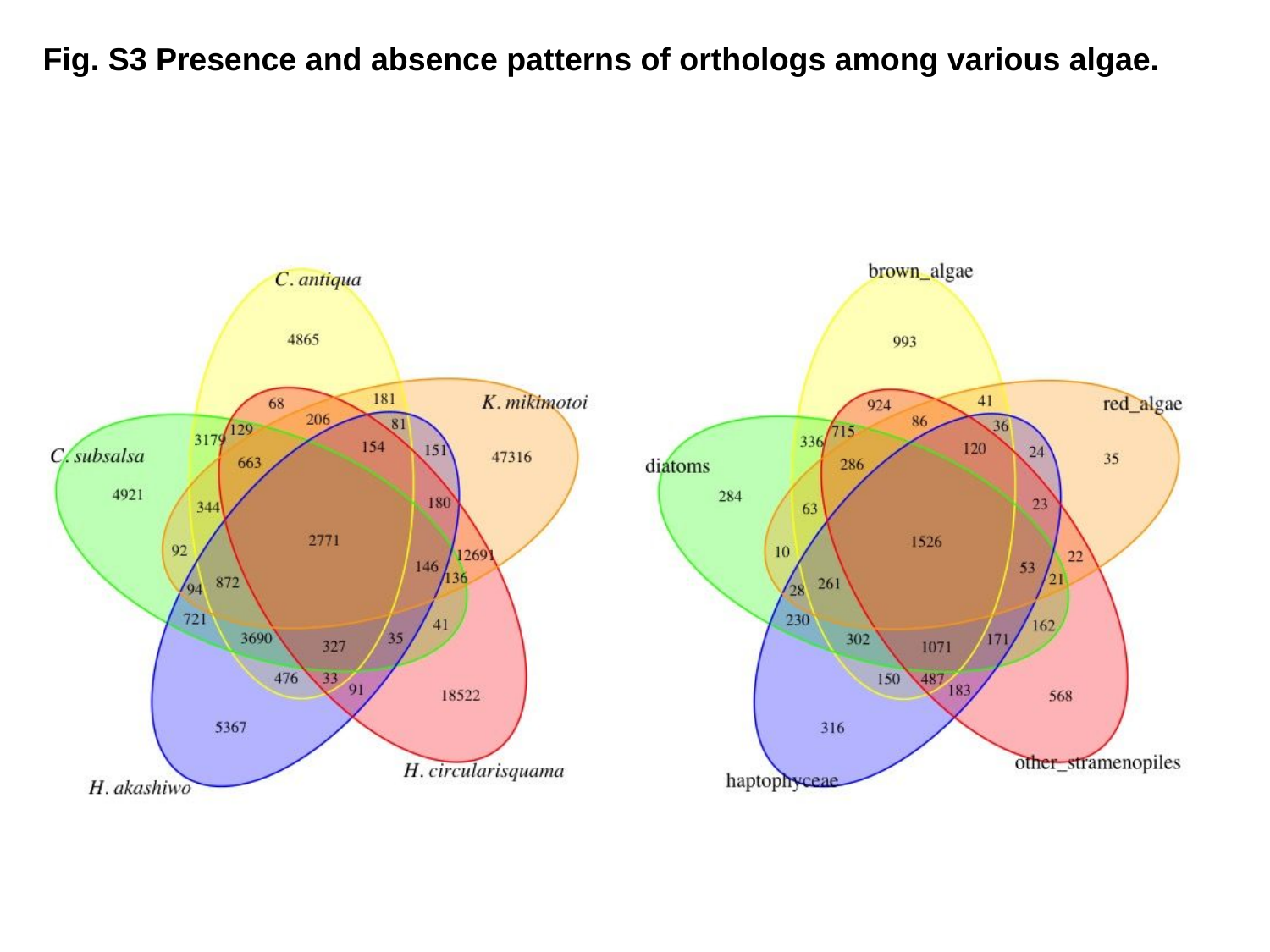

Fig. S3 Presence and absence patterns of orthologs among various algae.

## Slide 4
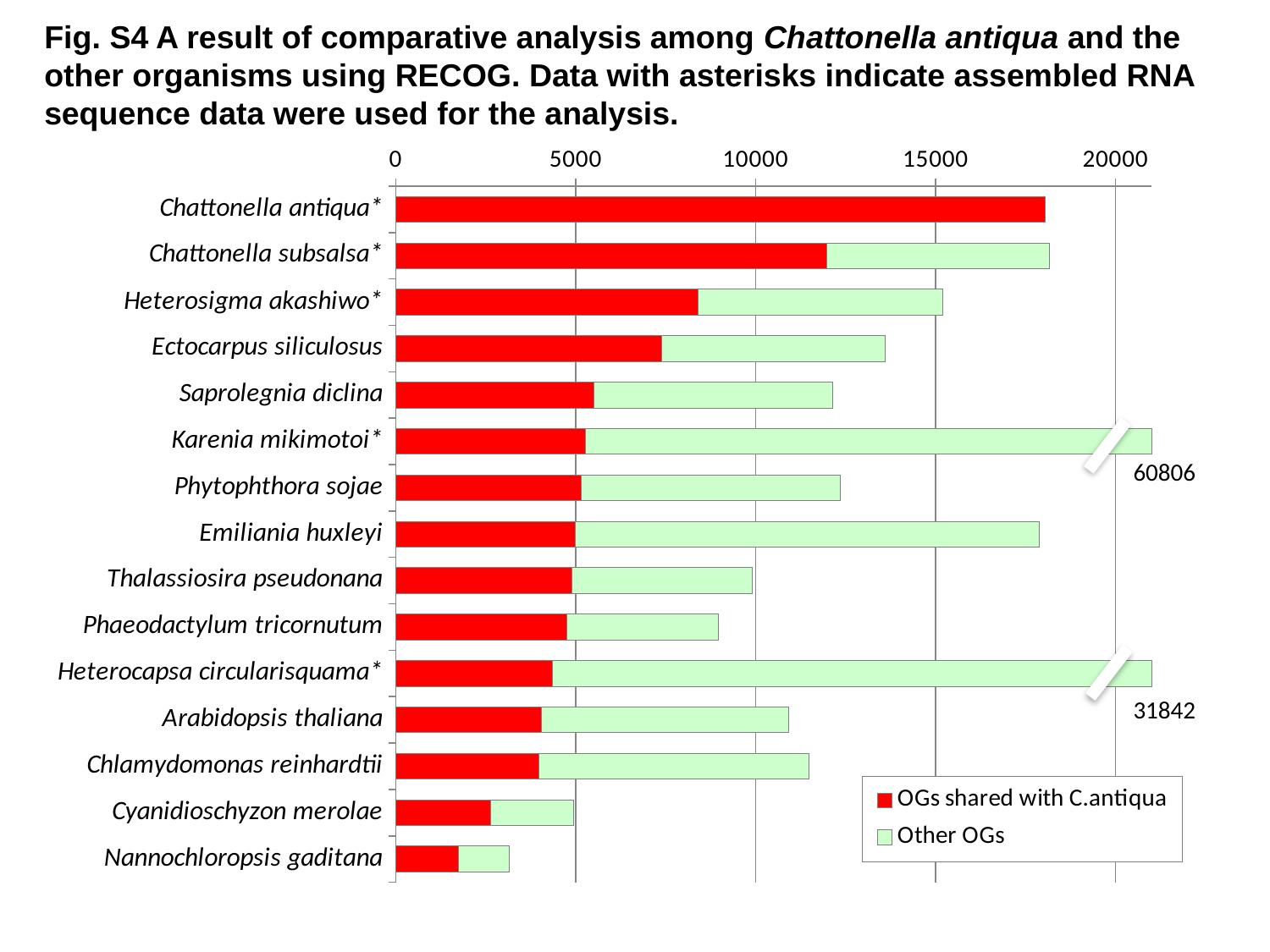

Fig. S4 A result of comparative analysis among Chattonella antiqua and the other organisms using RECOG. Data with asterisks indicate assembled RNA sequence data were used for the analysis.
### Chart
| Category | OGs shared with C.antiqua | Other OGs |
|---|---|---|
| Chattonella antiqua* | 18039.0 | 0.0 |
| Chattonella subsalsa* | 11975.0 | 6186.0 |
| Heterosigma akashiwo* | 8404.0 | 6785.0 |
| Ectocarpus siliculosus | 7397.0 | 6205.0 |
| Saprolegnia diclina | 5511.0 | 6630.0 |
| Karenia mikimotoi* | 5272.0 | 60806.0 |
| Phytophthora sojae | 5162.0 | 7202.0 |
| Emiliania huxleyi | 4981.0 | 12902.0 |
| Thalassiosira pseudonana | 4907.0 | 5009.0 |
| Phaeodactylum tricornutum | 4748.0 | 4220.0 |
| Heterocapsa circularisquama* | 4351.0 | 31842.0 |
| Arabidopsis thaliana | 4038.0 | 6878.0 |
| Chlamydomonas reinhardtii | 3989.0 | 7506.0 |
| Cyanidioschyzon merolae | 2635.0 | 2313.0 |
| Nannochloropsis gaditana | 1754.0 | 1401.0 |60806
31842

## Slide 5
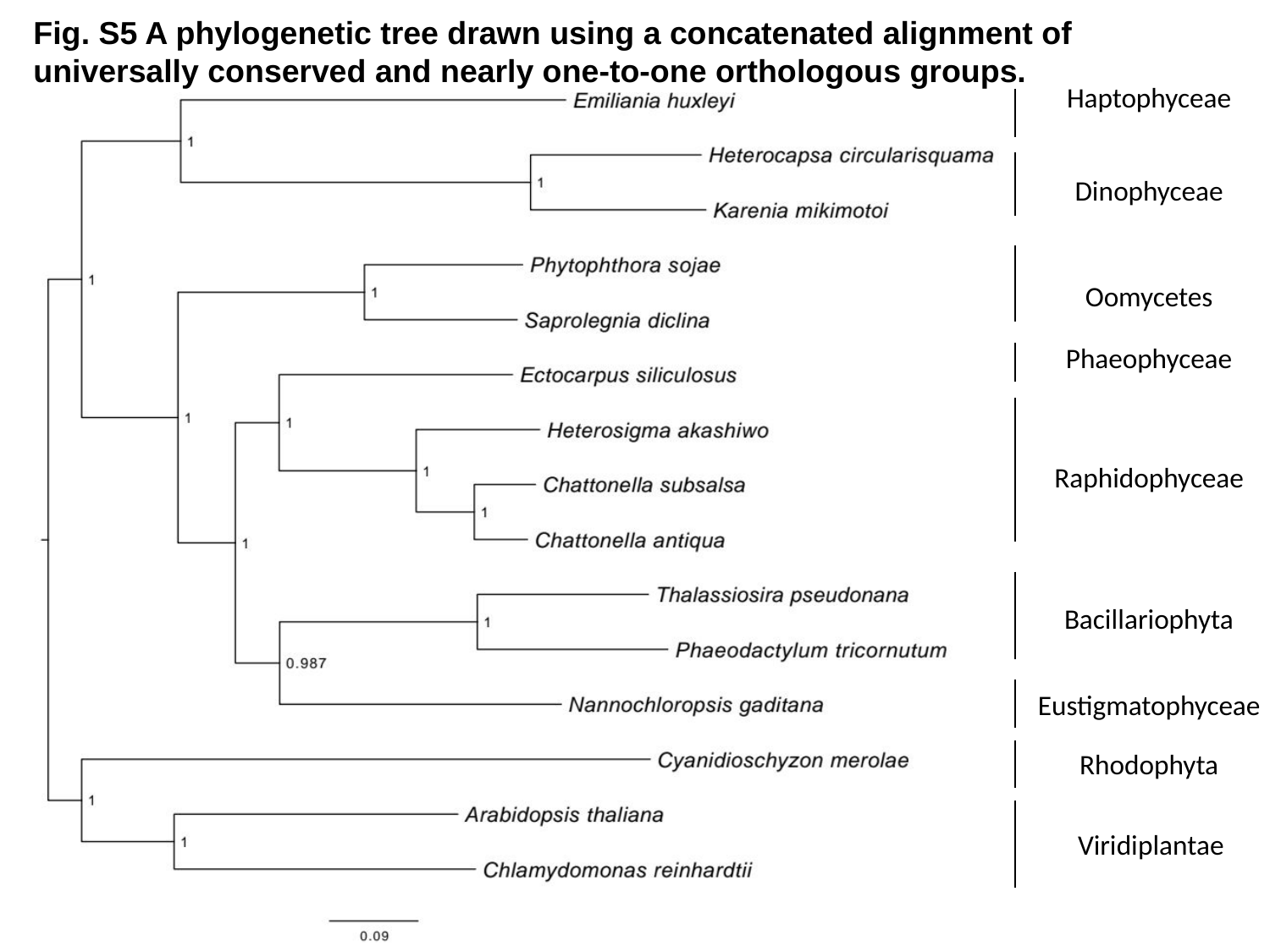

Fig. S5 A phylogenetic tree drawn using a concatenated alignment of universally conserved and nearly one-to-one orthologous groups.
Haptophyceae
Dinophyceae
Oomycetes
Phaeophyceae
Raphidophyceae
Bacillariophyta
Eustigmatophyceae
Rhodophyta
Viridiplantae

## Slide 6
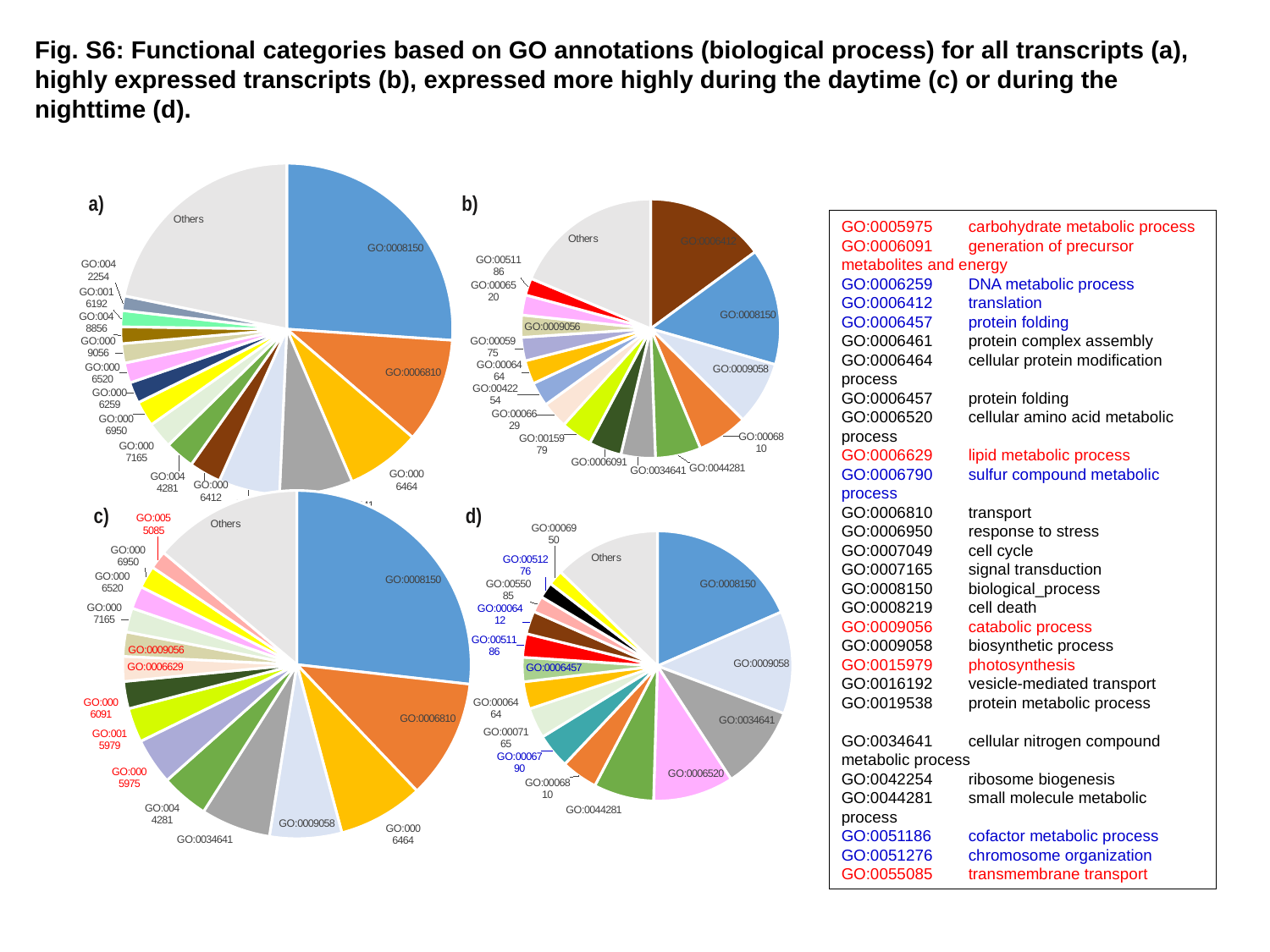

Fig. S6: Functional categories based on GO annotations (biological process) for all transcripts (a), highly expressed transcripts (b), expressed more highly during the daytime (c) or during the nighttime (d).
### Chart
| Category | |
|---|---|
| GO:0006412 | 59.1891233766234 |
| GO:0008150 | 57.8333333333333 |
| GO:0009058 | 31.48411814793389 |
| GO:0006810 | 25.1208333333333 |
| GO:0044281 | 22.528373015873 |
| GO:0034641 | 17.0552960927961 |
| GO:0006091 | 16.20064745196319 |
| GO:0015979 | 15.5194444444444 |
| GO:0006629 | 13.1970238095238 |
| GO:0042254 | 11.9815476190476 |
| GO:0006464 | 11.7299875563033 |
| GO:0005975 | 11.6173141186299 |
| GO:0009056 | 10.9609038622197 |
| GO:0006520 | 9.98333333333333 |
| GO:0051186 | 8.4313492063492 |
| Others | 74.16737129829235 |
### Chart
| Category | |
|---|---|
| GO:0008150 | 1016.18452380952 |
| GO:0006810 | 398.566159263442 |
| GO:0006464 | 282.447987842089 |
| GO:0034641 | 278.2079124378039 |
| GO:0009058 | 232.056297283093 |
| GO:0006412 | 121.445075757576 |
| GO:0044281 | 112.410443722944 |
| GO:0007165 | 99.52708611309868 |
| GO:0006950 | 95.9173144536921 |
| GO:0006259 | 80.57223044413449 |
| GO:0006520 | 76.35 |
| GO:0009056 | 73.4890025609067 |
| GO:0048856 | 63.49624258274149 |
| GO:0016192 | 61.239352663609 |
| GO:0042254 | 55.5713235294118 |
| Others | 848.5190475359361 |a)
b)
GO:0005975	carbohydrate metabolic process
GO:0006091	generation of precursor metabolites and energy
GO:0006259	DNA metabolic process
GO:0006412	translation
GO:0006457	protein folding
GO:0006461	protein complex assembly
GO:0006464	cellular protein modification process
GO:0006457	protein folding
GO:0006520	cellular amino acid metabolic process
GO:0006629	lipid metabolic process
GO:0006790	sulfur compound metabolic process
GO:0006810	transport
GO:0006950	response to stress
GO:0007049	cell cycle
GO:0007165	signal transduction
GO:0008150	biological_process
GO:0008219	cell death
GO:0009056	catabolic process
GO:0009058	biosynthetic process
GO:0015979	photosynthesis
GO:0016192	vesicle-mediated transport
GO:0019538	protein metabolic process
GO:0034641	cellular nitrogen compound metabolic process
GO:0042254	ribosome biogenesis
GO:0044281	small molecule metabolic process
GO:0051186	cofactor metabolic process
GO:0051276	chromosome organization
GO:0055085	transmembrane transport
### Chart
| Category | |
|---|---|
| GO:0008150 | 71.83333333333329 |
| GO:0006810 | 29.75 |
| GO:0006464 | 21.26144688644689 |
| GO:0009058 | 17.940873015873 |
| GO:0034641 | 17.34160561660561 |
| GO:0044281 | 11.8249389499389 |
| GO:0005975 | 11.5742063492063 |
| GO:0015979 | 8.51944444444444 |
| GO:0006091 | 6.7813492063492 |
| GO:0006629 | 6.2 |
| GO:0009056 | 6.064682539682539 |
| GO:0007165 | 5.97702659659182 |
| GO:0006520 | 5.66666666666667 |
| GO:0006950 | 5.554700854700849 |
| GO:0055085 | 4.5 |
| Others | 37.20972554016032 |
### Chart
| Category | |
|---|---|
| GO:0008150 | 19.0 |
| GO:0009058 | 12.6469696969697 |
| GO:0034641 | 10.4083333333333 |
| GO:0006520 | 9.91666666666667 |
| GO:0044281 | 7.43106060606061 |
| GO:0006810 | 4.5 |
| GO:0006790 | 4.28333333333333 |
| GO:0007165 | 3.75 |
| GO:0006464 | 3.375 |
| GO:0006457 | 3.0 |
| GO:0051186 | 2.90833333333333 |
| GO:0006412 | 2.833333333333329 |
| GO:0055085 | 2.0 |
| GO:0051276 | 1.95833333333333 |
| GO:0006950 | 1.875 |
| Others | 13.11363636363636 |c)
d)

## Slide 7
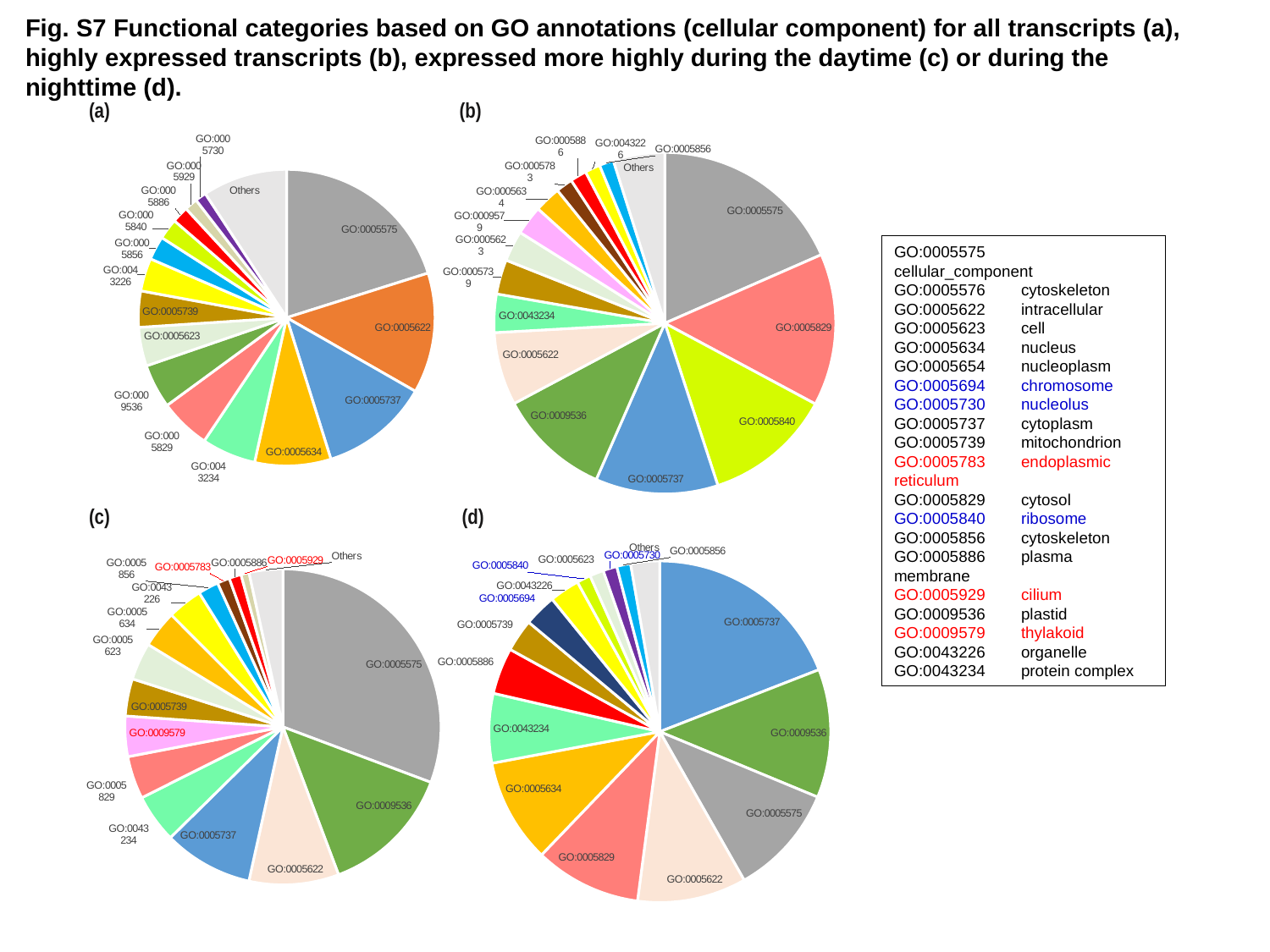

Fig. S7 Functional categories based on GO annotations (cellular component) for all transcripts (a), highly expressed transcripts (b), expressed more highly during the daytime (c) or during the nighttime (d).
(a)
(b)
### Chart
| Category | |
|---|---|
| GO:0005575 | 68.0 |
| GO:0005829 | 53.1952380952381 |
| GO:0005840 | 44.7595238095238 |
| GO:0005737 | 42.8333333333333 |
| GO:0009536 | 39.1785714285714 |
| GO:0005622 | 25.5 |
| GO:0043234 | 13.4261904761905 |
| GO:0005739 | 12.0690476190476 |
| GO:0005623 | 10.6666666666667 |
| GO:0009579 | 10.2261904761905 |
| GO:0005634 | 9.23571428571429 |
| GO:0005783 | 5.83333333333333 |
| GO:0005886 | 5.73571428571429 |
| GO:0043226 | 5.33333333333333 |
| GO:0005856 | 5.0 |
| Others | 18.00714285714286 |
### Chart
| Category | |
|---|---|
| GO:0005575 | 630.0 |
| GO:0005622 | 410.85 |
| GO:0005737 | 372.122014652015 |
| GO:0005634 | 259.695494505494 |
| GO:0043234 | 184.592424242424 |
| GO:0005829 | 173.3741452991449 |
| GO:0009536 | 150.154761904762 |
| GO:0005623 | 132.416666666667 |
| GO:0005739 | 123.385714285714 |
| GO:0043226 | 112.472161172161 |
| GO:0005856 | 80.736013986014 |
| GO:0005840 | 70.77738095238097 |
| GO:0005886 | 56.28769841269849 |
| GO:0005929 | 44.37070707070709 |
| GO:0005730 | 37.7710317460317 |
| Others | 288.9937851037853 |GO:0005575	cellular_component
GO:0005576	cytoskeleton
GO:0005622	intracellular
GO:0005623	cell
GO:0005634	nucleus
GO:0005654	nucleoplasm
GO:0005694	chromosome
GO:0005730	nucleolus
GO:0005737	cytoplasm
GO:0005739	mitochondrion
GO:0005783	endoplasmic reticulum
GO:0005829	cytosol
GO:0005840	ribosome
GO:0005856	cytoskeleton
GO:0005886	plasma membrane
GO:0005929	cilium
GO:0009536	plastid
GO:0009579	thylakoid
GO:0043226	organelle
GO:0043234	protein complex
(c)
(d)
### Chart
| Category | |
|---|---|
| GO:0005575 | 68.5 |
| GO:0009536 | 30.2261904761905 |
| GO:0005622 | 20.5 |
| GO:0005737 | 20.4166666666667 |
| GO:0043234 | 11.0833333333333 |
| GO:0005829 | 9.75952380952381 |
| GO:0009579 | 9.226190476190467 |
| GO:0005739 | 8.50952380952381 |
| GO:0005623 | 8.5 |
| GO:0005634 | 8.476190476190478 |
| GO:0043226 | 8.0 |
| GO:0005856 | 4.66666666666667 |
| GO:0005783 | 2.833333333333329 |
| GO:0005886 | 2.75 |
| GO:0005929 | 1.86666666666667 |
| Others | 7.685714285714279 |
### Chart
| Category | |
|---|---|
| GO:0005737 | 14.5 |
| GO:0009536 | 9.25 |
| GO:0005575 | 8.0 |
| GO:0005622 | 7.83333333333333 |
| GO:0005829 | 7.66666666666667 |
| GO:0005634 | 7.5 |
| GO:0043234 | 5.0 |
| GO:0005886 | 3.333333333333329 |
| GO:0005739 | 2.333333333333329 |
| GO:0005694 | 2.333333333333329 |
| GO:0043226 | 2.16666666666667 |
| GO:0005840 | 1.0 |
| GO:0005623 | 1.0 |
| GO:0005730 | 1.0 |
| GO:0005856 | 1.0 |
| Others | 2.083333333333333 |

## Slide 8
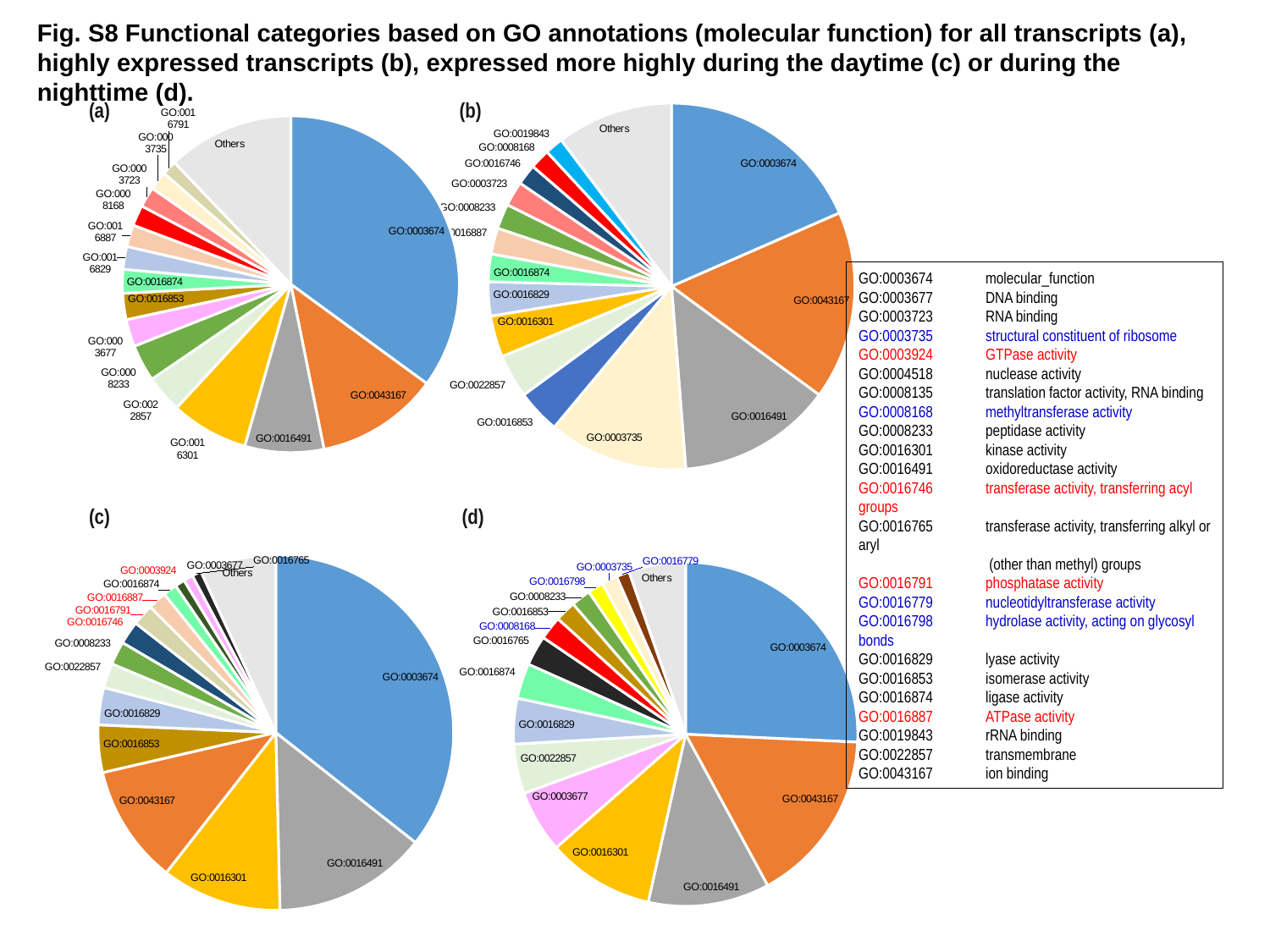

Fig. S8 Functional categories based on GO annotations (molecular function) for all transcripts (a), highly expressed transcripts (b), expressed more highly during the daytime (c) or during the nighttime (d).
(a)
(b)
### Chart
| Category | |
|---|---|
| GO:0003674 | 87.95833333333329 |
| GO:0043167 | 79.43333333333338 |
| GO:0016491 | 65.1666666666667 |
| GO:0003735 | 58.66666666666669 |
| GO:0016853 | 18.4 |
| GO:0022857 | 18.33333333333329 |
| GO:0016301 | 17.40833333333329 |
| GO:0016829 | 14.3333333333333 |
| GO:0016874 | 11.6166666666667 |
| GO:0016887 | 10.9833333333333 |
| GO:0008233 | 10.5 |
| GO:0003723 | 10.4166666666667 |
| GO:0016746 | 8.700000000000001 |
| GO:0008168 | 8.4 |
| GO:0019843 | 7.66666666666667 |
| Others | 49.01666666666664 |
### Chart
| Category | |
|---|---|
| GO:0003674 | 1644.74572649573 |
| GO:0043167 | 553.4184787434781 |
| GO:0016491 | 356.054761904762 |
| GO:0016301 | 349.324891774892 |
| GO:0022857 | 168.533333333333 |
| GO:0008233 | 166.2 |
| GO:0003677 | 122.655555555556 |
| GO:0016853 | 118.766666666667 |
| GO:0016874 | 106.7 |
| GO:0016829 | 102.828571428571 |
| GO:0016887 | 98.36880341880338 |
| GO:0008168 | 92.8166666666667 |
| GO:0003723 | 91.65 |
| GO:0003735 | 85.0 |
| GO:0016791 | 65.83333333333329 |
| Others | 570.1032106782106 |GO:0003674	molecular_function
GO:0003677	DNA binding
GO:0003723	RNA binding
GO:0003735	structural constituent of ribosome
GO:0003924	GTPase activity
GO:0004518	nuclease activity
GO:0008135	translation factor activity, RNA binding
GO:0008168	methyltransferase activity
GO:0008233	peptidase activity
GO:0016301	kinase activity
GO:0016491	oxidoreductase activity
GO:0016746	transferase activity, transferring acyl groups
GO:0016765	transferase activity, transferring alkyl or aryl
	 (other than methyl) groups
GO:0016791	phosphatase activity
GO:0016779	nucleotidyltransferase activity
GO:0016798	hydrolase activity, acting on glycosyl bonds
GO:0016829	lyase activity
GO:0016853	isomerase activity
GO:0016874	ligase activity
GO:0016887	ATPase activity
GO:0019843	rRNA binding
GO:0022857	transmembrane
GO:0043167	ion binding
(c)
(d)
### Chart
| Category | |
|---|---|
| GO:0003674 | 127.166666666667 |
| GO:0016491 | 50.0 |
| GO:0016301 | 39.04166666666669 |
| GO:0043167 | 38.6583333333333 |
| GO:0016853 | 15.4 |
| GO:0016829 | 12.0833333333333 |
| GO:0022857 | 8.0 |
| GO:0008233 | 7.58333333333333 |
| GO:0016746 | 7.5 |
| GO:0016791 | 7.0 |
| GO:0016887 | 6.08333333333333 |
| GO:0016874 | 4.5 |
| GO:0003924 | 3.16666666666667 |
| GO:0003677 | 3.08333333333333 |
| GO:0016765 | 3.0 |
| Others | 24.73333333333332 |
### Chart
| Category | |
|---|---|
| GO:0003674 | 35.0 |
| GO:0043167 | 22.25 |
| GO:0016491 | 15.5 |
| GO:0016301 | 13.6666666666667 |
| GO:0003677 | 8.0 |
| GO:0022857 | 6.33333333333333 |
| GO:0016829 | 5.83333333333333 |
| GO:0016874 | 4.5 |
| GO:0016765 | 3.833333333333329 |
| GO:0008168 | 3.0 |
| GO:0016853 | 2.66666666666667 |
| GO:0008233 | 2.5 |
| GO:0016798 | 2.0 |
| GO:0003735 | 2.0 |
| GO:0016779 | 1.66666666666667 |
| Others | 7.249999999999992 |

## Slide 9
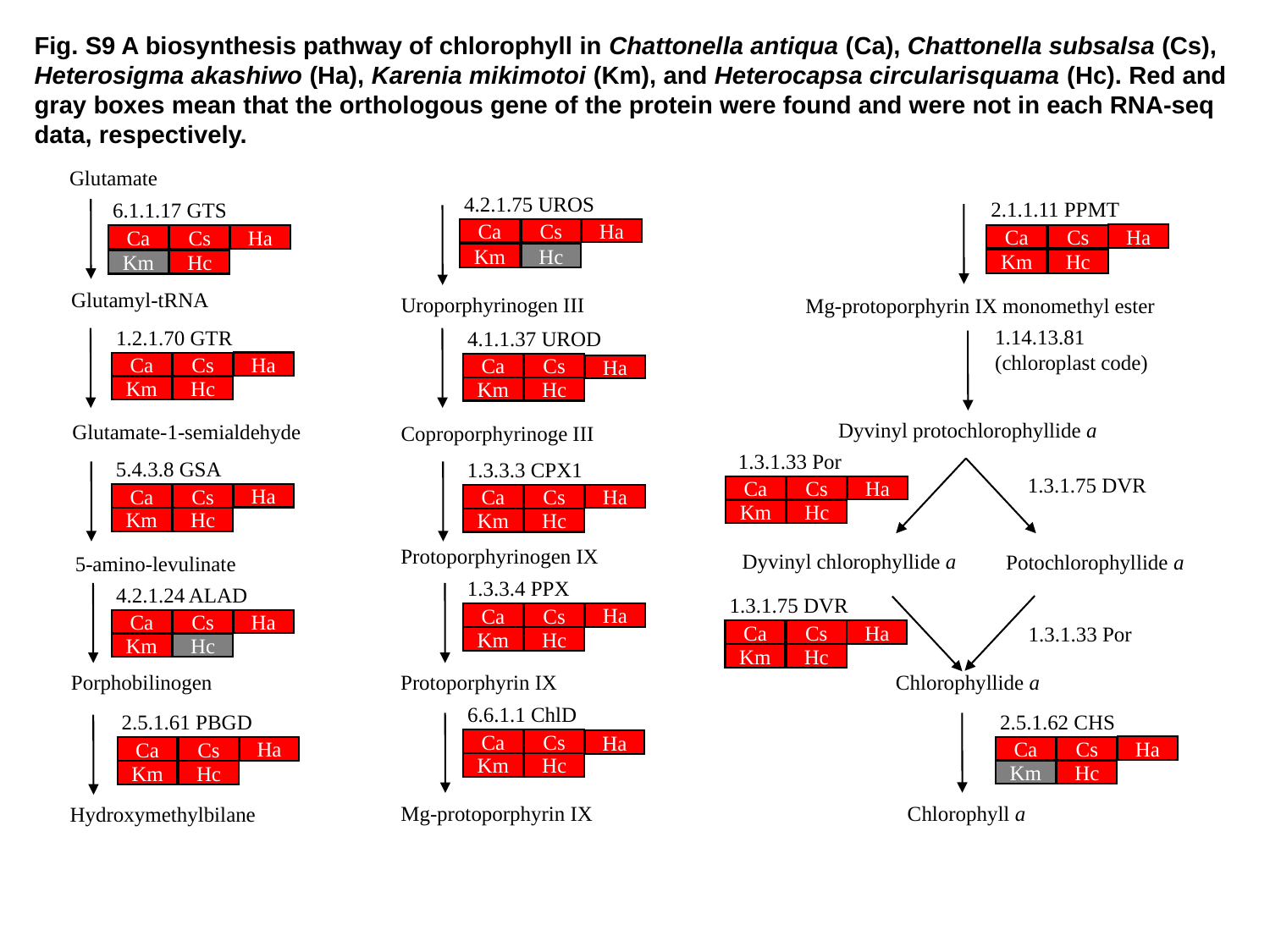

Fig. S9 A biosynthesis pathway of chlorophyll in Chattonella antiqua (Ca), Chattonella subsalsa (Cs), Heterosigma akashiwo (Ha), Karenia mikimotoi (Km), and Heterocapsa circularisquama (Hc). Red and gray boxes mean that the orthologous gene of the protein were found and were not in each RNA-seq data, respectively.
Glutamate
4.2.1.75 UROS
Ha
Ca
Cs
Km
Hc
2.1.1.11 PPMT
Ha
Ca
Cs
Km
Hc
6.1.1.17 GTS
Ha
Ca
Cs
Km
Hc
Mg-protoporphyrin IX monomethyl ester
Dyvinyl protochlorophyllide a
Dyvinyl chlorophyllide a
Potochlorophyllide a
Chlorophyllide a
Chlorophyll a
Uroporphyrinogen III
Coproporphyrinoge III
Protoporphyrinogen IX
Protoporphyrin IX
Mg-protoporphyrin IX
Glutamyl-tRNA
1.14.13.81
(chloroplast code)
1.2.1.70 GTR
Ha
Ca
Cs
Km
Hc
4.1.1.37 UROD
Ca
Cs
Ha
Km
Hc
Glutamate-1-semialdehyde
1.3.1.33 Por
Ha
Ca
Cs
Km
Hc
5.4.3.8 GSA
Ha
Ca
Cs
Km
Hc
1.3.3.3 CPX1
Ha
Ca
Cs
Km
Hc
1.3.1.75 DVR
5-amino-levulinate
1.3.3.4 PPX
Ha
Ca
Cs
Km
Hc
4.2.1.24 ALAD
Ha
Ca
Cs
Km
Hc
1.3.1.75 DVR
Ha
Ca
Cs
Km
Hc
1.3.1.33 Por
Porphobilinogen
6.6.1.1 ChlD
Ca
Cs
Ha
Km
Hc
2.5.1.62 CHS
Ha
Ca
Cs
Km
Hc
2.5.1.61 PBGD
Ha
Ca
Cs
Km
Hc
Hydroxymethylbilane

## Slide 10
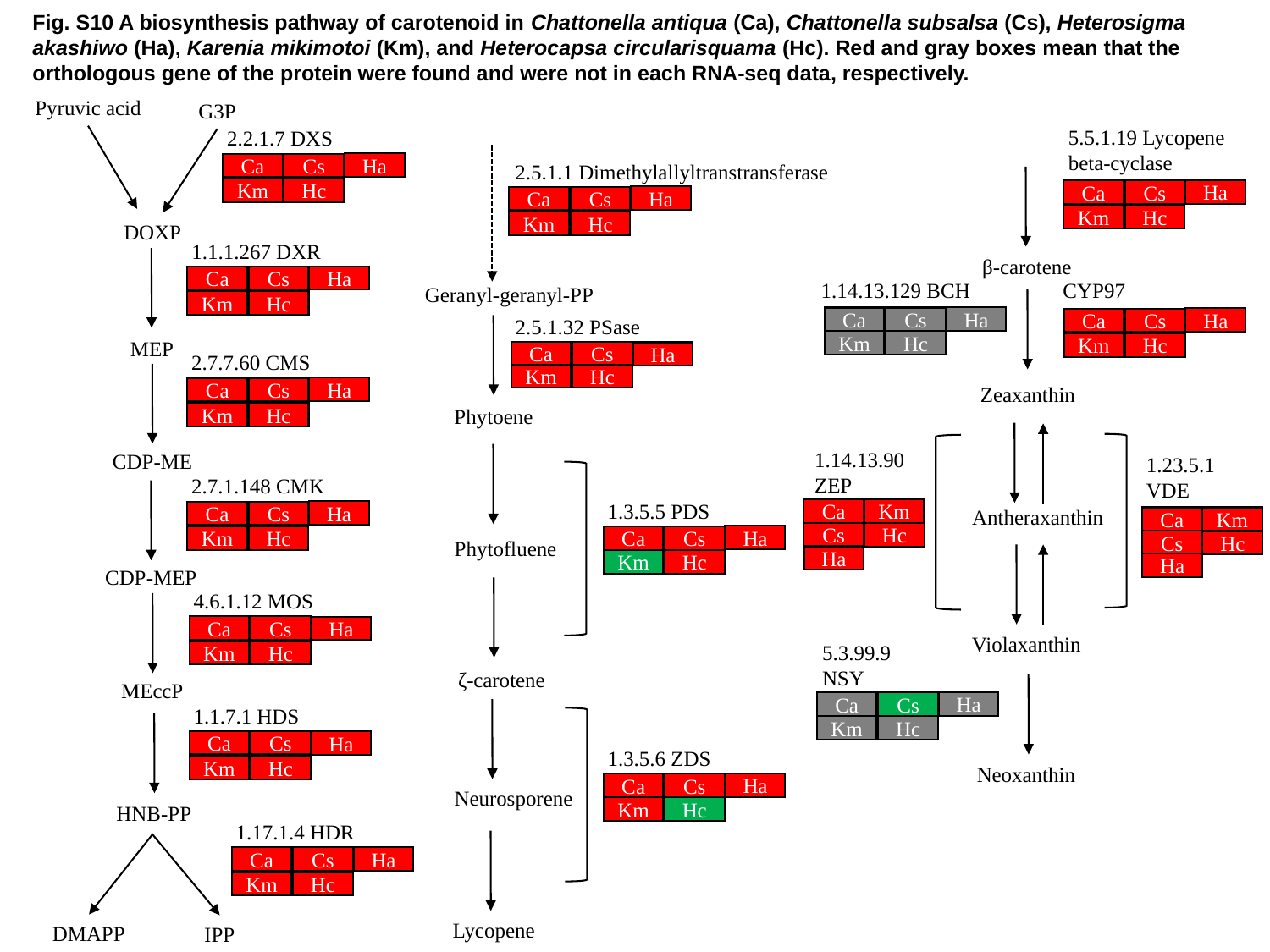

Fig. S10 A biosynthesis pathway of carotenoid in Chattonella antiqua (Ca), Chattonella subsalsa (Cs), Heterosigma akashiwo (Ha), Karenia mikimotoi (Km), and Heterocapsa circularisquama (Hc). Red and gray boxes mean that the orthologous gene of the protein were found and were not in each RNA-seq data, respectively.
Pyruvic acid
G3P
5.5.1.19 Lycopene
beta-cyclase
Ha
Ca
Cs
Km
Hc
2.2.1.7 DXS
Ha
Ca
Cs
Km
Hc
2.5.1.1 Dimethylallyltranstransferase
Ha
Ca
Cs
Km
Hc
β-carotene
Zeaxanthin
Antheraxanthin
Violaxanthin
Neoxanthin
DOXP
1.1.1.267 DXR
Ha
Ca
Cs
Km
Hc
1.14.13.129 BCH
CYP97
Geranyl-geranyl-PP
2.5.1.32 PSase
Ca
Cs
Ha
Km
Hc
Ha
Ca
Cs
Km
Hc
Ha
Ca
Cs
Km
Hc
MEP
2.7.7.60 CMS
Ha
Ca
Cs
Km
Hc
Phytoene
1.14.13.90
ZEP
Ca
Km
Cs
Hc
Ha
CDP-ME
1.23.5.1
VDE
Ca
Km
Cs
Hc
Ha
2.7.1.148 CMK
Ha
Ca
Cs
Km
Hc
1.3.5.5 PDS
Ha
Ca
Cs
Km
Hc
Phytofluene
CDP-MEP
4.6.1.12 MOS
Ca
Cs
Ha
Km
Hc
5.3.99.9
NSY
Ha
Ca
Cs
Km
Hc
ζ-carotene
MEccP
1.1.7.1 HDS
Ca
Cs
Ha
Km
Hc
1.3.5.6 ZDS
Ha
Ca
Cs
Km
Hc
Neurosporene
HNB-PP
1.17.1.4 HDR
Ha
Ca
Cs
Km
Hc
Lycopene
DMAPP
IPP

## Slide 11
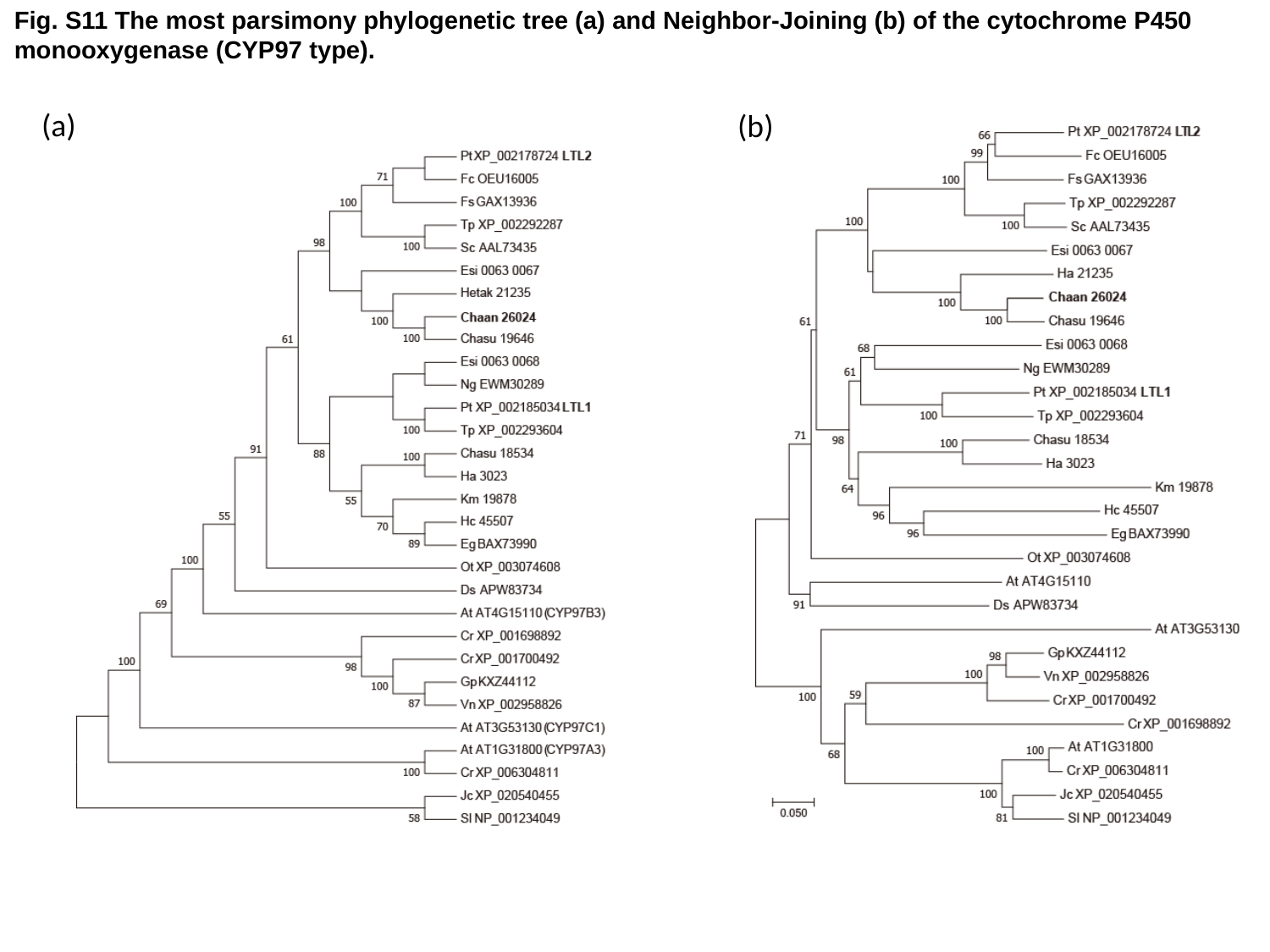

Fig. S11 The most parsimony phylogenetic tree (a) and Neighbor-Joining (b) of the cytochrome P450 monooxygenase (CYP97 type).
(a)
(b)

## Slide 12
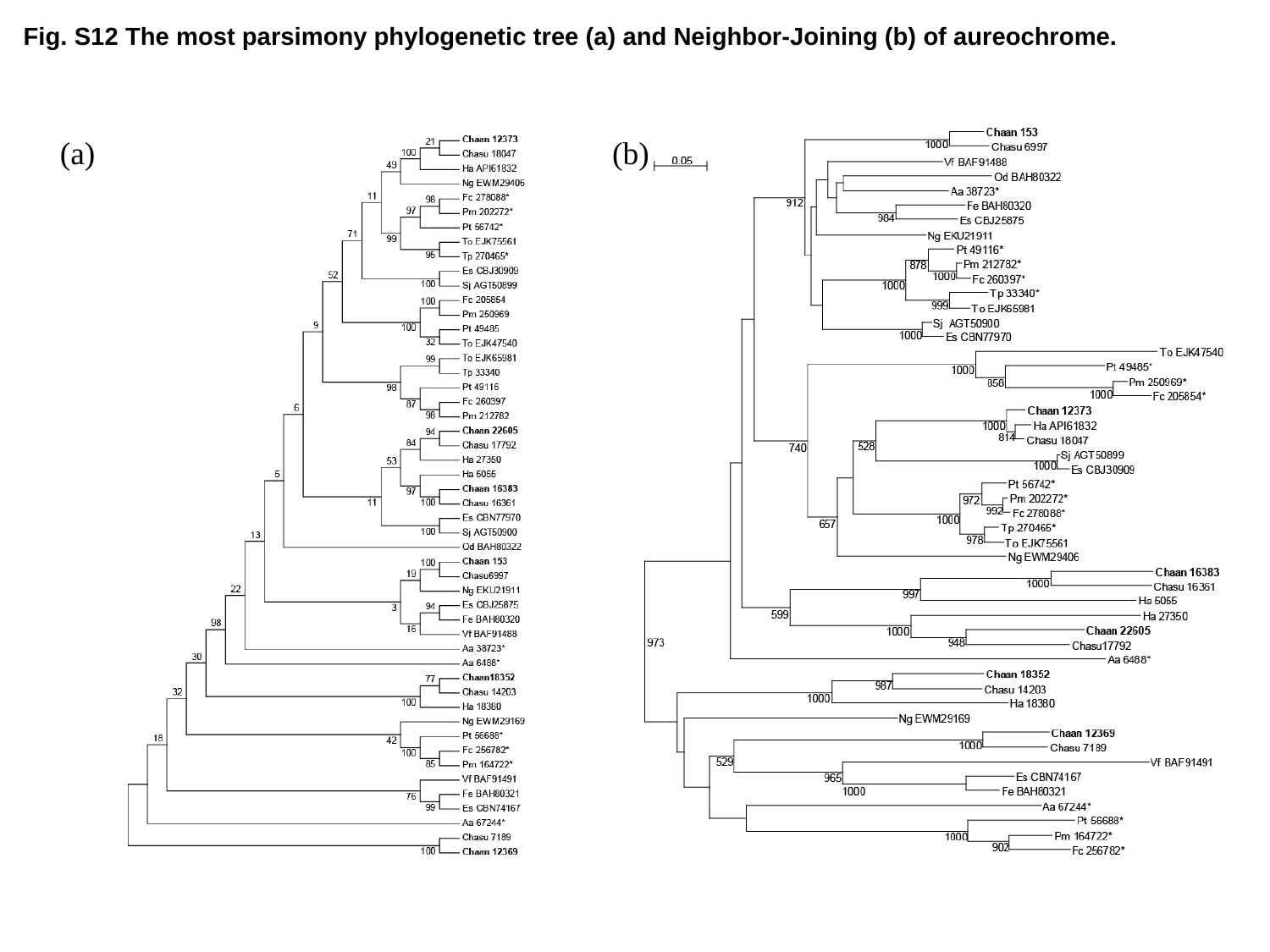

Fig. S12 The most parsimony phylogenetic tree (a) and Neighbor-Joining (b) of aureochrome.
(a)
(b)

## Slide 13
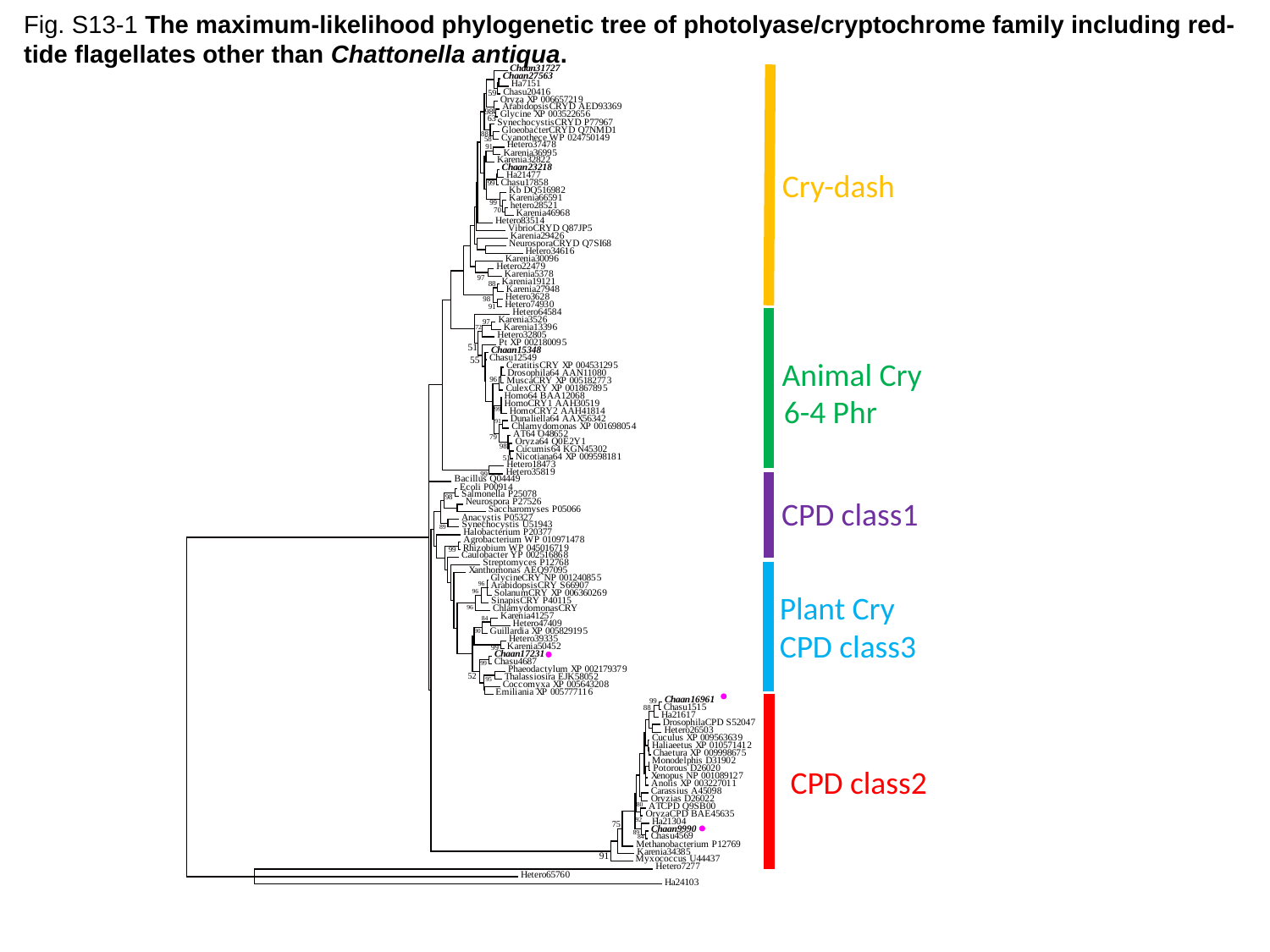

Fig. S13-1 The maximum-likelihood phylogenetic tree of photolyase/cryptochrome family including red-tide flagellates other than Chattonella antiqua.
Cry-dash
Animal Cry
6-4 Phr
CPD class1
Plant Cry
CPD class3
CPD class2

## Slide 14
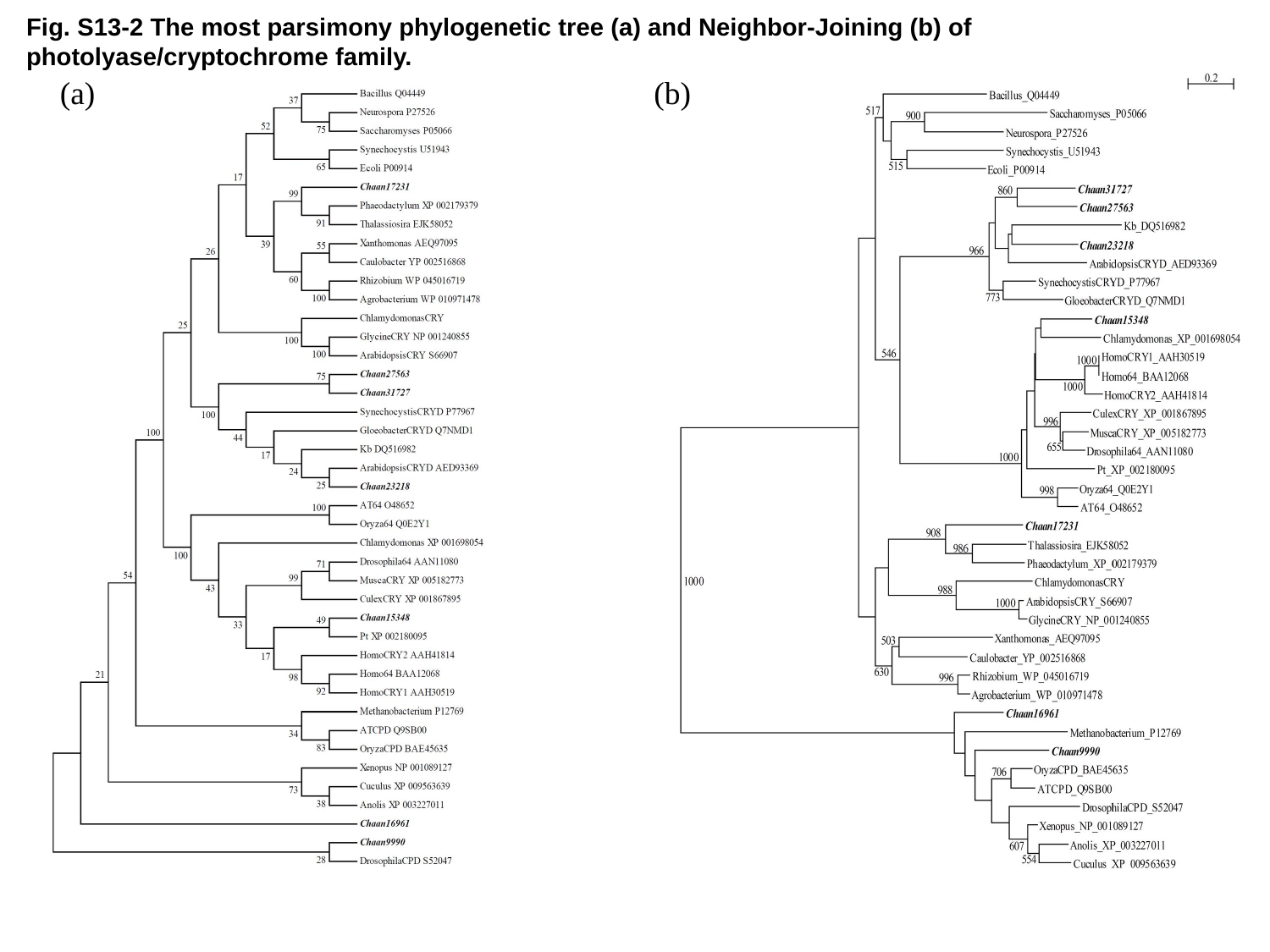

Fig. S13-2 The most parsimony phylogenetic tree (a) and Neighbor-Joining (b) of photolyase/cryptochrome family.
(a)
(b)

## Slide 15
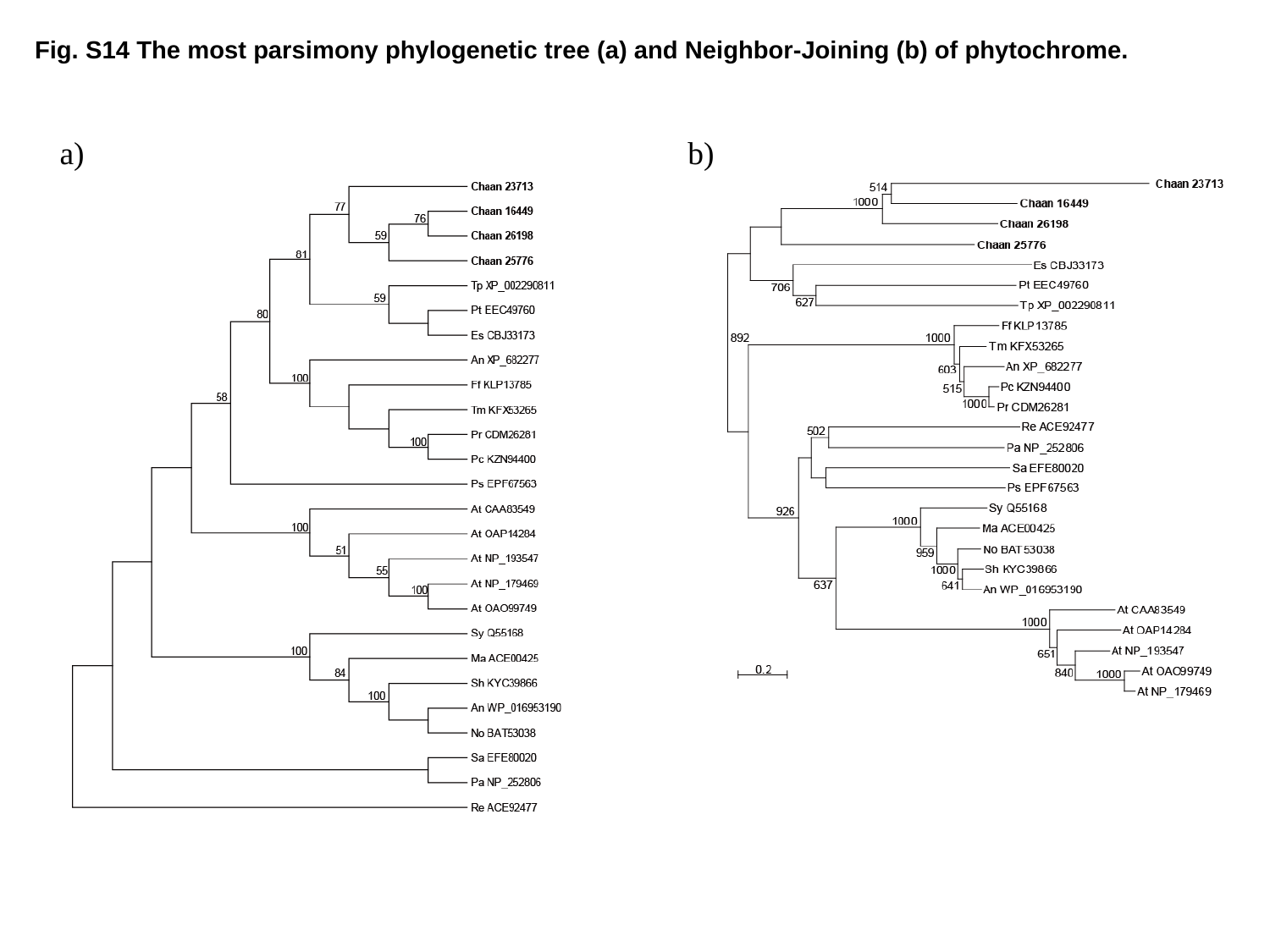

Fig. S14 The most parsimony phylogenetic tree (a) and Neighbor-Joining (b) of phytochrome.
a)
b)

## Slide 16
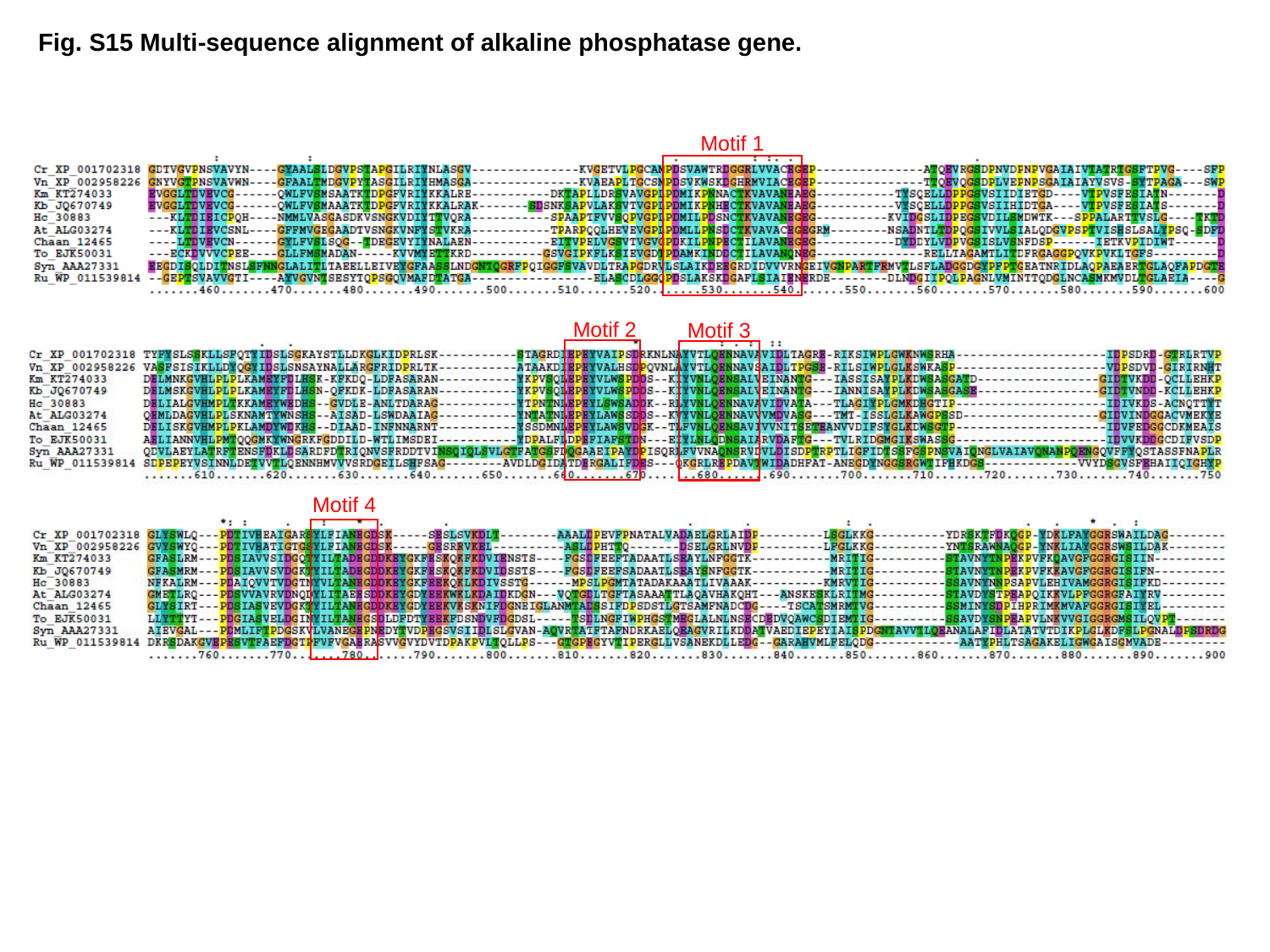

Fig. S15 Multi-sequence alignment of alkaline phosphatase gene.
Motif 1
Motif 2
Motif 3
Motif 4

## Slide 17
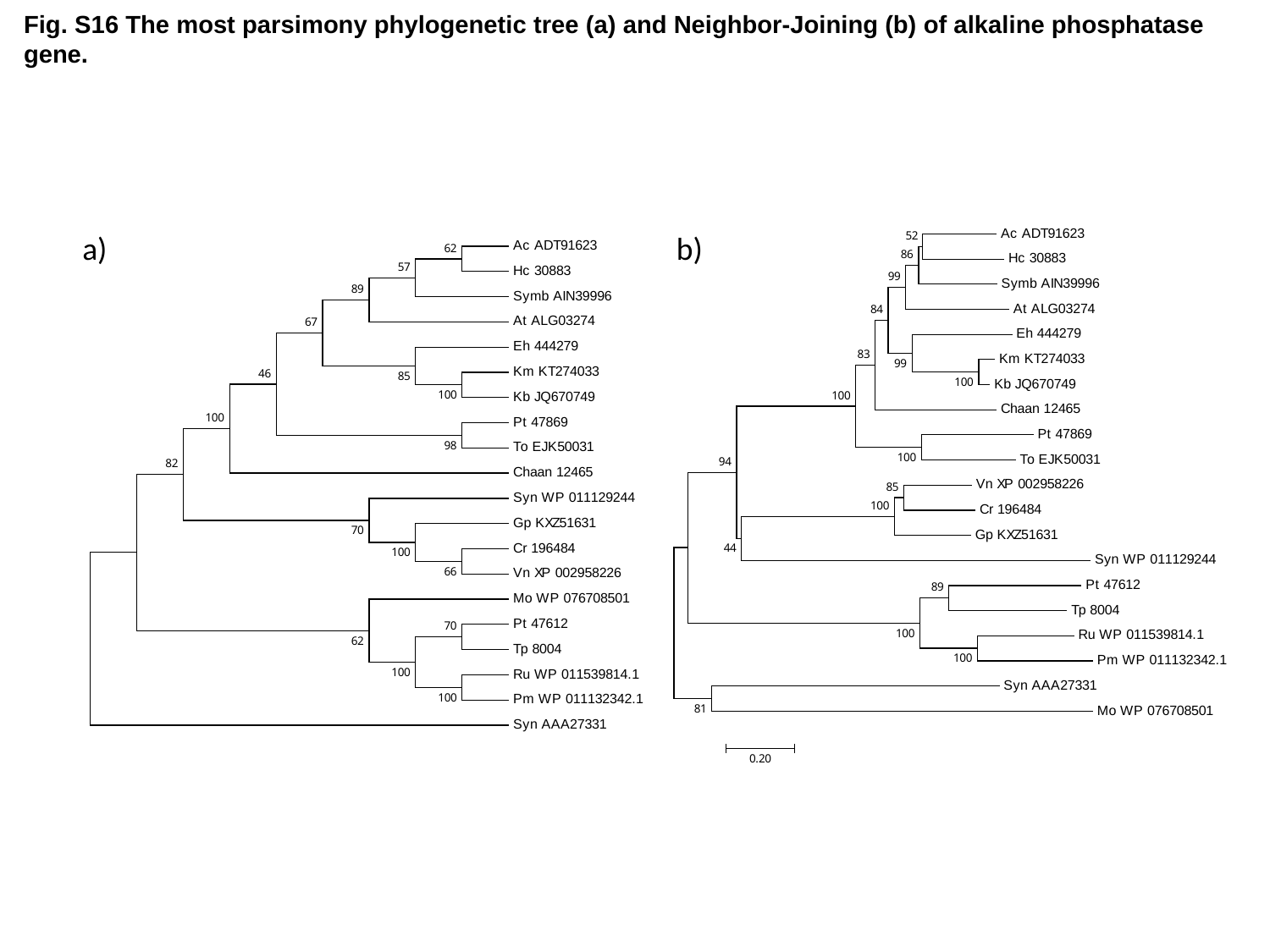

Fig. S16 The most parsimony phylogenetic tree (a) and Neighbor-Joining (b) of alkaline phosphatase gene.
a)
b)

## Slide 18
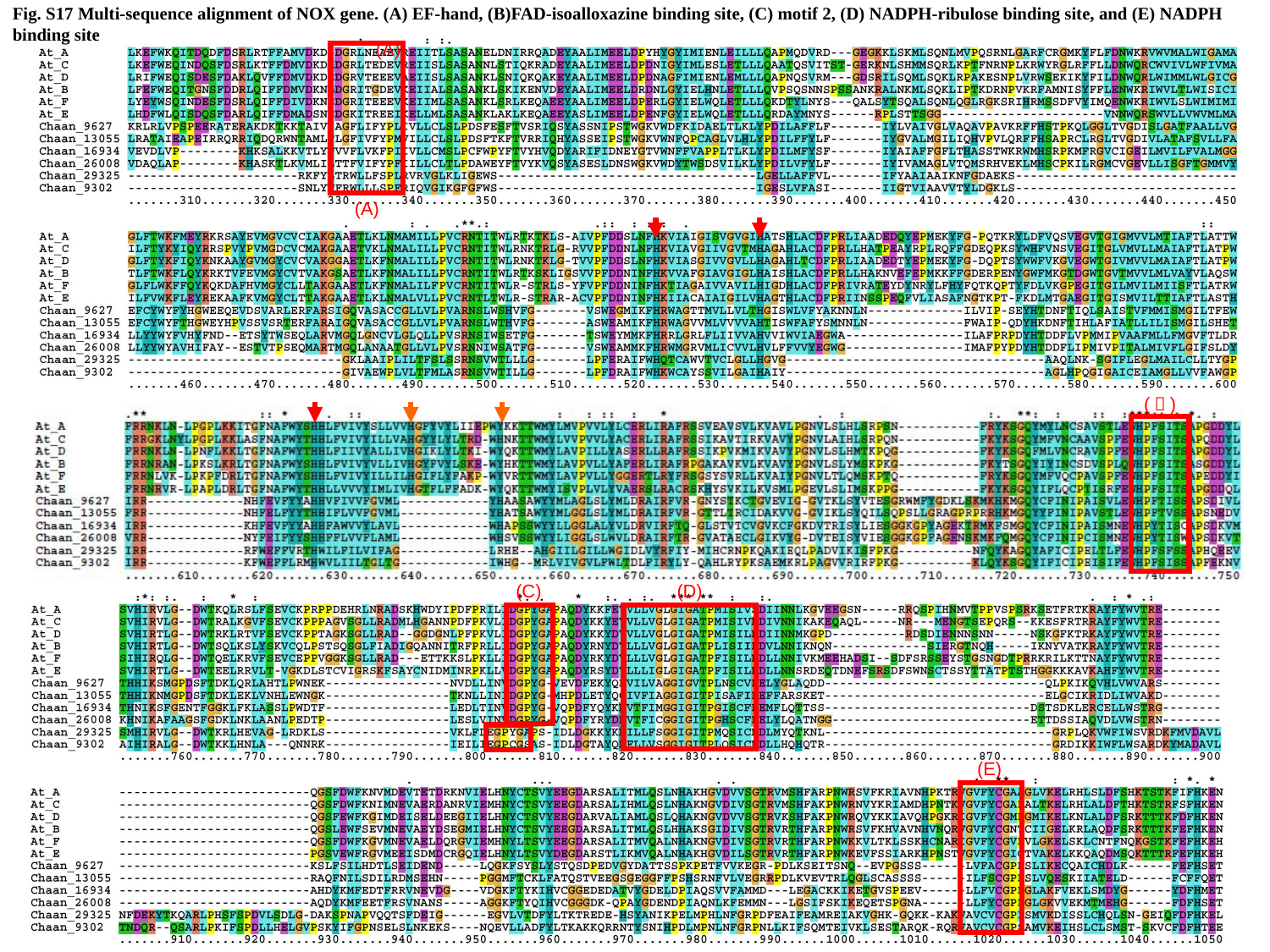

Fig. S17 Multi-sequence alignment of NOX gene. (A) EF-hand, (B)FAD-isoalloxazine binding site, (C) motif 2, (D) NADPH-ribulose binding site, and (E) NADPH binding site
(A)
(A)
(Ｂ)
(D)
(C)
(E)

## Slide 19
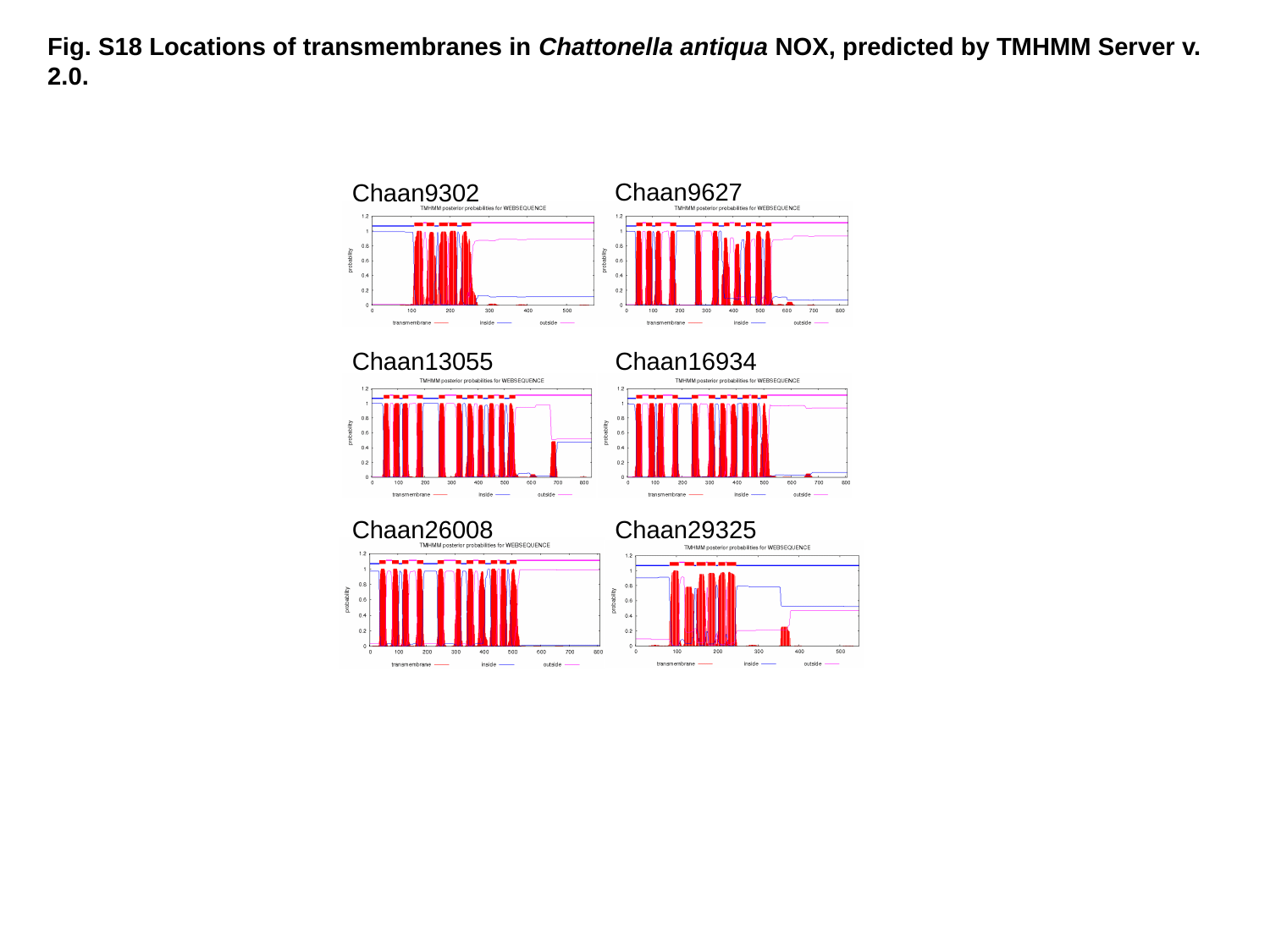

Fig. S18 Locations of transmembranes in Chattonella antiqua NOX, predicted by TMHMM Server v. 2.0.
Chaan9627
Chaan9302
Chaan13055
Chaan16934
Chaan26008
Chaan29325
